# Pyruvate kinase deficiency modifies sickle hemoglobin carrier and sickle cell disease phenotypes in mice

**DOI:** 10.1172/jci.insight.195682

**Published:** 2026-01-08

**Authors:** Xunde Wang, Meghann Smith, Sayuri Kamimura, Quan Li, Niharika Shah, Martha Quezado, Luis E.F. Almeida, Sebastian Vogel, Mickias B. Tegegn, Kevin Y. Sun, Rafael Villasmil, Chengyu Liu, William A. Eaton, Swee Lay Thein, Zenaide M.N. Quezado

**Affiliations:** 1 Sickle Cell Branch, National Heart Lung and Blood Institute,; 2 Department of Perioperative Medicine, National Institutes of Health Clinical Center,; 3 Laboratory of Chemical Physics, National Institute of Diabetes and Digestive and Kidney Diseases,; 4 Laboratory of Pathology, National Cancer Institute,; 5 Flow Cytometry Core Facility, National Eye Institute, and; 6 National Heart Lung and Blood Institute, NIH, Bethesda, Maryland, USA.

**Keywords:** Genetics, Hematology, Bioenergetics, Glucose metabolism, Mouse models

## Abstract

Growing evidence indicates that *PKLR*, the gene for pyruvate kinase (PK), is a genetic modifier of the sickle cell phenotype. Coinheritance of specific *PKLR* variants is associated with increased pain-related hospitalization and can trigger sickle cell disease (SCD) phenotypes in asymptomatic carriers. PK deficiency disrupts RBC glycolysis, leading to ATP deficits and accumulation of 2,3-diphosphoglycerate, which exacerbates sickling in SCD. Using CRISPR-Cas9, we generated null mutations in *Pklr* [*Pklr^(13ntdel/13ntdel)^* or *Pklr^(246ntdel/246ntdel)^*] specific for the RBC isoform (PKR) in Townes mice that were homozygous (SS) or heterozygous (AS) for the human sickle globin gene, or homozygous for human hemoglobin A (AA, controls), to investigate the effect of PKR deficiency on the sickle phenotype in mice. PKR-deficient AA and AS mice developed severe anemia, reticulocytosis, and substantial spleen and liver iron deposits. Unlike what is observed in humans, PKR deficiency in AS and SS mice surprisingly decreased sickling, but it was also associated with increased extramedullary hematopoiesis and mitochondrial retention in mature RBCs. These results demonstrate the differential effect of *Pklr* mutations on the phenotype of both AS and SS mouse models, offering insights into the complex role of PKR deficiency in SCD pathology.

## Abstract 2

*Pklr* mutations associated with pyruvate kinase deficiency modify the phenotype of sickle cell trait and sickle cell disease in mouse models

## Graphical abstract

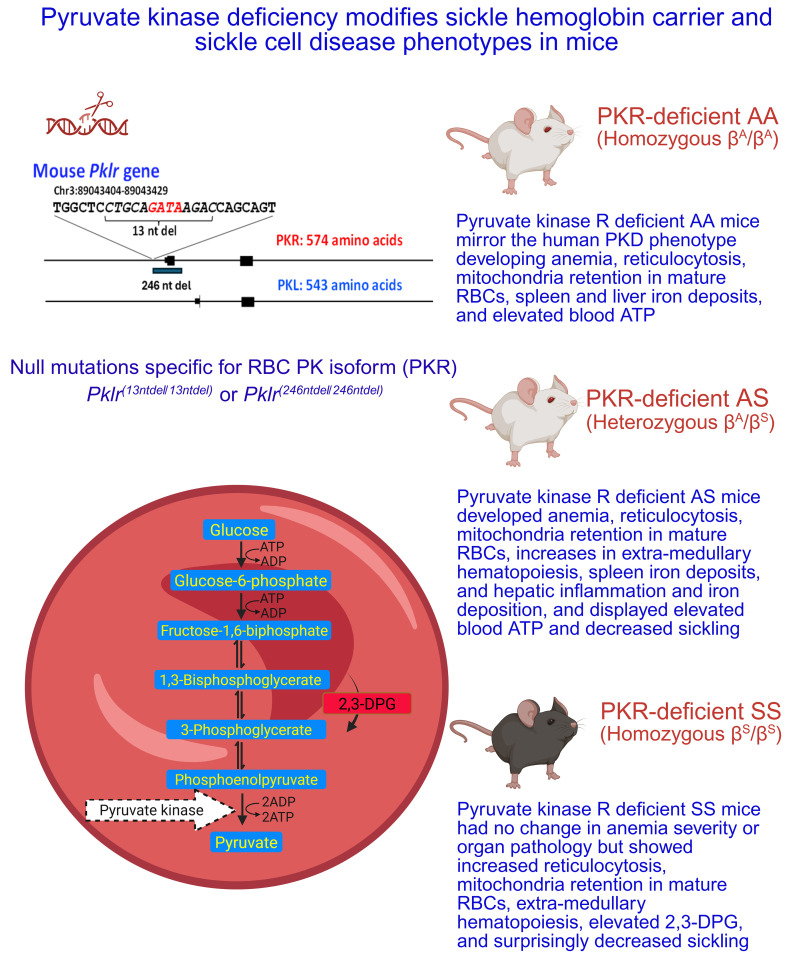

## Introduction

Sickle cell disease (SCD) affects 1 in 350 African American newborns in the United States and nearly 8 million people worldwide (1, 2). The disease is caused by inheritance of sickle hemoglobin (HbS) that polymerizes when deoxygenated, to form fibers that stiffen and deforms (sickles) RBCs. RBC sickling triggers downstream effects including hemolytic anemia, recurrent micro-vaso-occlusion and acute pain episodes, that underlie the chronic inflammation and vascular endothelial injury, leading to multisystem organ damage and, ultimately, reduced life expectancy (3, 4). SCD is caused by a single mutation in *HBB*, yet its clinical manifestations vary widely due to both environmental factors and coinherited genetic modifiers. Two established modifiers include genetic variants that alter fetal hemoglobin (HbF) levels and α-thalassemia status (5–7). However, these modifiers do not fully account for the observed phenotypic heterogeneity (5). Therefore, identifying additional genetic modifiers would contribute to the development of biomarkers, prognostic tools, and therapeutic strategies to mitigate disease severity (8–10).

Emerging evidence identifies *PKLR*, the gene encoding for pyruvate kinase (PK) liver and RBC isoenzymes, PKL and PKR, respectively, as a genetic modifier of sickle genotypes (11). Pyruvate kinase is a critical enzyme in the glycolysis pathway, the sole source of ATP in RBCs. A deficiency in PK activity leads to a metabolic block resulting in an ATP deficit and accumulation of the upstream substrates including 2,3-diphosphoglycerate (2,3-DPG), a key allosteric modulator of hemoglobin’s oxygen affinity (12–14). These metabolic alterations are detrimental in sickle RBCs, as 2,3-DPG promotes sickling by stabilizing the HbS polymers, while ATP depletion reduces RBC hydration and compromises membrane stability (12–15). The clinical relevance of *PKLR* in the sickle phenotype is illustrated by findings from a candidate gene association study, where we identified 7 *PKLR* intronic variants associated with hospitalization rates for acute sickle pain in adult and pediatric patients with SCD (16). Additional evidence of *PKLR*’s role in sickle cell pathophysiology comes from individuals with sickle cell trait (SCT), which is characteristically clinically benign, who nevertheless had typical SCD. These individuals were found to have coinheritance of *PKLR* mutations associated with reduced PK levels (17, 18). These findings then prompted the ongoing clinical trials of PK activators that provide proof-of-principle that enhancing PK activity increases ATP and reduces 2,3-DPG levels yielding promising clinical benefits in patients with SCD (19–23). Both PK activators, mitapivat and etavopivat, increased ATP/2,3-DPG ratio, reduced sickling, and improved hemoglobin levels with concomitant reduction in hemolytic markers in patients with SCD (19–21). In a phase 2, randomized double-blind, placebo-controlled global trial, patients with SCD who received mitapivat had a higher hemoglobin response rate (increase in average hemoglobin concentration ≥ 1.0 g/dL from baseline from week 10 through week 12), with improvement in markers of hemolysis and erythropoiesis compared with patients who received placebo. Mitapivat-treated patients also had a reduction in annualized rate of sickle cell pain crises (22). Collectively, these clinical trials support continuing development of PK activators as disease-modifying therapy in SCD.

To further investigate how PKR deficiency influences the sickle phenotype, we introduced loss-of-function *Pklr* mutations specific for the PKR isoform into the humanized Townes model of SCD. Using CRISPR-Cas9, we generated Townes mice homozygous (β^S^/β^S^, SS) and heterozygous (β^A^/β^S^, AS) for the human β^S^-globin gene, alongside control mice homozygous for the human β^A^-globin gene (β^A^/β^A^, AA) with coinheritance of loss-of-function mutations in *Pklr* specific for the PKR isoform. We hypothesize that PKR deficiency would accentuate the sickle phenotype in the humanized mouse models of SCD as observed in humans.

## Results

### AA, AS, and SS Townes mice with Pklr-null mutations are viable, lack PKR expression, and have intact expression of other PK isoforms.

Townes AA (β^A^/β^A^, controls), AS (β^A^/β^S^, HbS carrier), and SS (β^S^/β^S^, SCD) mice with coinheritance of *Pklr^(WT/13ntdel)^*, *Pklr^(13ntdel/13ntdel)^*, *Pklr^(WT/246ntdel)^,* and *Pklr^(246ntdel/246ntdel)^* mutations were viable. However, those with *Pklr^(WT/246ntdel)^* and *Pklr^(246ntdel/246ntdel)^* mutations displayed decreased reproductive capacity; hence, some outcome measures for complete absence of PKR were assessed only in mice with coinheritance of *Pklr^(13ntdel/13ntdel)^*. We confirmed that mice with coinheritance of both *Pklr*-null mutant alleles lacked expression of PKR but retained expression of PKL (Supplemental Figure 1, A–D; supplemental material available online with this article; https://doi.org/10.1172/jci.insight.195682DS1) and of the pyruvate kinase muscle isoform (PKM; Supplemental Figure 2, A and B). Supplemental Tables 1 and 2 list mean ± SD for all outcome measurements and *P* values of comparisons examining the effects of coinheritance of *Pklr* null mutations [*Pklr^(13ntdel/13ntdel)^* or *Pklr^(246ntdel/246ntdel)^*] in AA, AS, and SS mice versus their respective *Pklr^(WT/WT)^* counterparts.

### Coinheritance of PKR deficiency [Pklr^(13ntdel/13ntdel)^ or Pklr^(246ntdel/246ntdel)^] differentially affects blood levels of ATP and 2,3-DPG in AA, AS, and SS mice.

**Figure 1:**
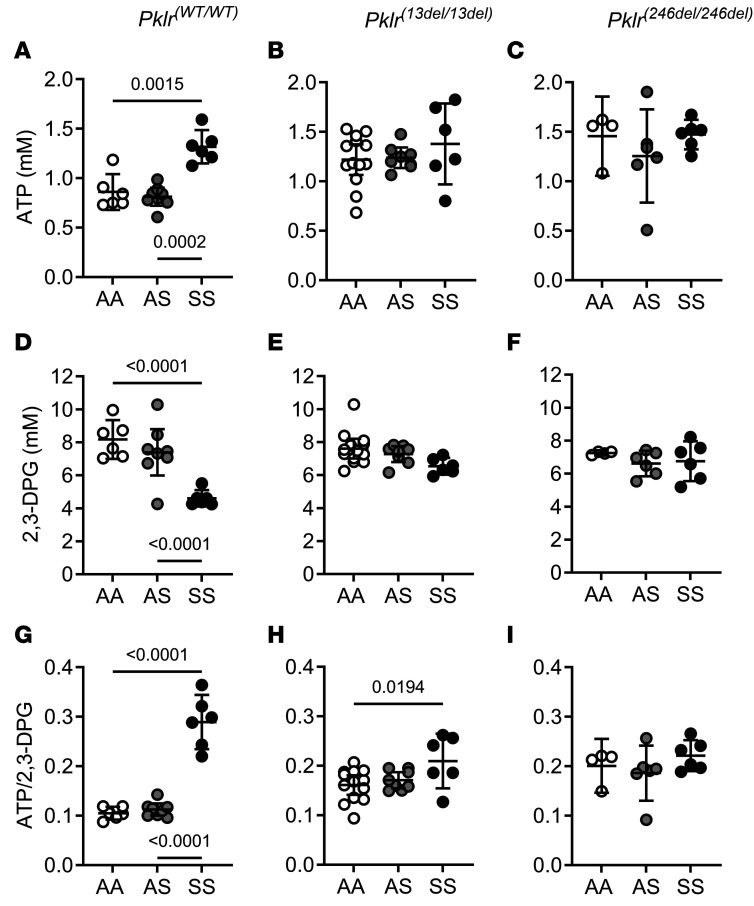
Coinheritance of *Pklr*-null mutations [*Pklr^(13ntdel/13ntdel)^* or *Pklr^(246ntdel/246ntdel)^*] specific for the RBC pyruvate kinase isoform (PKR) differentially alters ATP and 2,3-diphosphoglycerate (2,3-DPG) levels in AA, AS, and SS mice. Data are shown as scatter dot plots illustrating individual mouse measurements, with overlaid bars representing the least-squares mean ± 95% CI. Data were analyzed using a 2-way ANOVA, and *P* values were adjusted for multiple comparisons using the Tukey method. All experimental groups included balanced number of age- and sex-matched mice. SS*Pklr^(WT/WT)^* mice had higher ATP (**A**), lower 2,3-DPG (**D**), and higher ATP/2,3-DPG ratio (**G**) compared with AA*Pklr^(WT/WT)^* and AS*Pklr^(WT/WT)^* animals (all *P* ≤ 0.0015). (**A**–**C**) The effect of *Pklr* mutations on ATP levels varied according to sickle genotype (*P* = 0.005 for genotype-by-*Pklr* mutation interactions). PKR-deficient AA and AS mice [*Pklr^(13ntdel/13ntdel)^* or *Pklr^(246ntdel/246ntdel)^*] had higher ATP levels compared with AA*Pklr^(WT/WT)^* and AS*Pklr^(WT/WT)^* respectively (all *P* ≤ 0.0112, **A**–**C**). Conversely, PKR-deficient SS mice had similar blood ATP levels compared with SS*Pklr^(WT/WT)^* (*P* ≥ 0.7318, **A**–**C**). *Pklr* mutations also altered 2,3-DPG levels differentially depending on the sickle genotype (*P* < 0.0001 for genotype-by-*Pklr* mutation interactions). PKR-deficient [*Pklr^(13ntdel/13ntdel)^* or *Pklr^(246ntdel/246ntdel)^*] AA and AS mice had similar 2,3-DPG levels compared with AA*Pklr^(WT/WT)^* and AS*Pklr^(WT/WT)^*, respectively (all, *P* ≥ 0.6275, **D**–**F**). Conversely, PKR-deficient SS [SS*Pklr^(13ntdel/13ntdel)^* and SS*Pklr^(246ntdel/246ntdel)^*] mice had higher 2,3-DPG levels compared with SS*Pklr^(WT/WT)^* (*P* = 0.0145 and *P* = 0.0048, respectively; **D**–**F**). (**G**–**I**) As a result, PKR-deficient AA and AS mice [*Pklr^(13ntdel/13ntdel)^* or *Pklr^(246ntdel/246ntdel)^*] had higher ATP/2,3-DPG ratios compared with AA*Pklr^(WT/WT)^* and AS*Pklr^(WT/WT)^* (*P* = 0.0171 and *P* = 0.0007 for AA and *P* = 0.0134 and *P* = 0.0023 for AS, respectively; **G**–**I**). In contrast, PKR-deficient SS mice had lower ATP/2,3-DPG ratio compared with SS*Pklr^(WT/WT)^* (*P* = 0.0020 and *P* = 0.0123, respectively; **G**–**I**).

We measured ATP and 2,3-DPG blood levels as an indirect measure of PKR activity in AA, AS, and SS mice with and without *Pklr* mutations (Figure 1 and Supplemental Table 1). Consistent with our previous reports (24, 25), among *Pklr^(WT/WT)^*, SS*Pklr^(WT/WT)^* had higher blood ATP (Figure 1A), lower 2,3-DPG (Figure 1D), and higher ATP/2,3-DPG ratio (Figure 1G) compared with AA*Pklr^(WT/WT)^* (*P* = 0.0015, < 0.0001, and < 0.0001, respectively) and AS*Pklr^(WT/WT)^* animals (*P* = 0.0002, < 0.0001, and < 0.0001, respectively, Supplemental Table 1).

Coinheritance of PKR deficiency influenced ATP and 2,3-DPG levels in AA and AS mice in a similar manner, yet it affected SS mice in a significantly different pattern (sickle genotype-by-*Pklr* mutation interactions *P* = 0.005 for ATP and *P* < 0.0001 for 2,3-DPG). PKR-deficient AA and AS mice had elevated ATP, similar 2,3-DPG, and higher ATP/2,3-DPG ratios compared with their WT counterparts [AA*Pklr^(WT/WT)^* and AS*Pklr^(WT/WT)^*] respectively (Figure 1, A–I, and Supplemental Table 1). Conversely, PKR-deficient SS mice had similar ATP, higher 2,3-DPG, and lower ATP/2,3-DPG ratio compared with SS*Pklr^(WT/WT)^* (Figure 1 and Supplemental Table 1).

### Coinheritance of PKR deficiency [Pklr^(13ntdel/13ntdel)^ or Pklr^(246ntdel/246ntdel)^] differentially affects blood cell counts and hematologic indices in AA, AS, and SS Townes mice.

**Figure 2:**
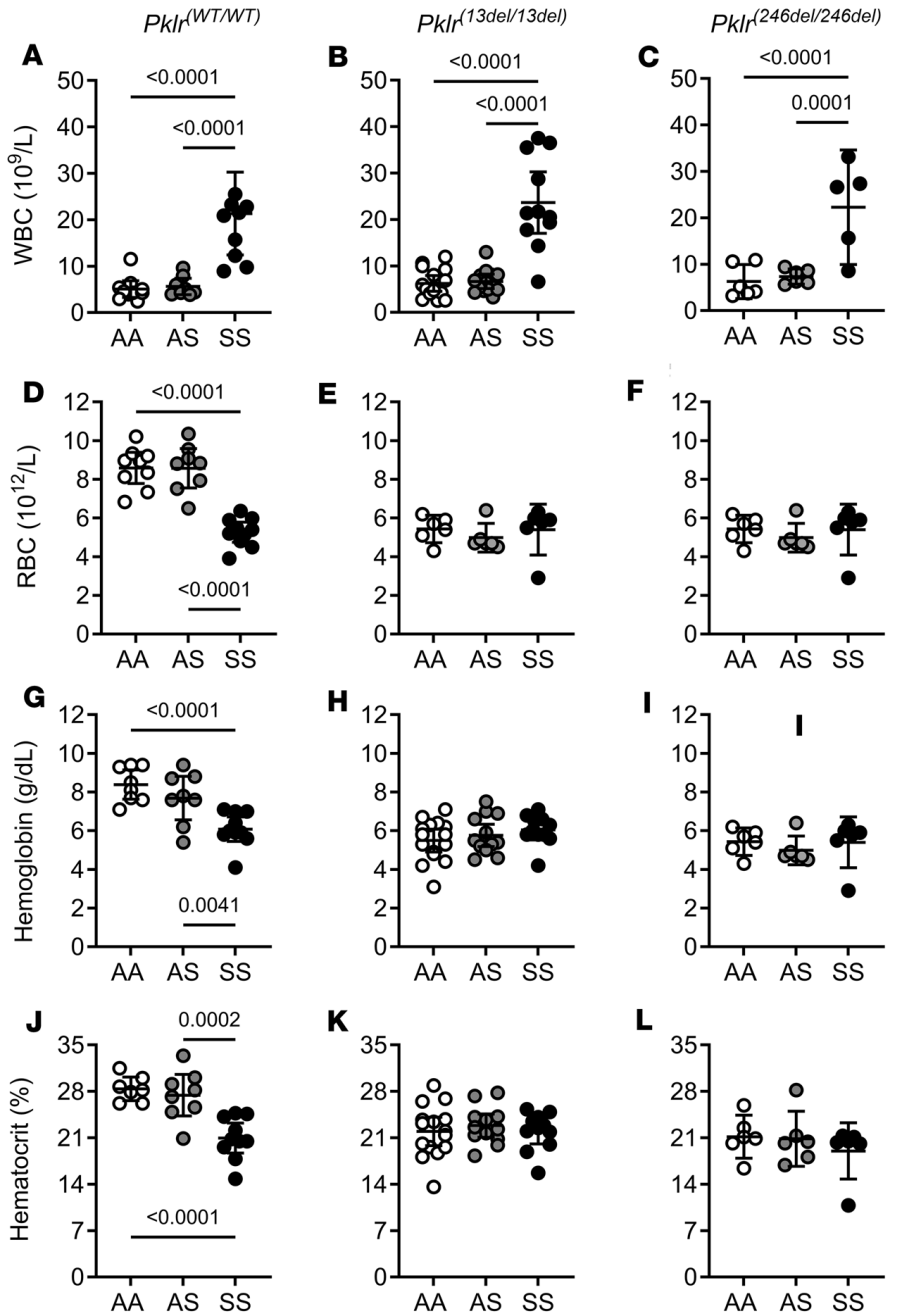
Coinheritance of *Pklr*-null mutations [*Pklr^(13ntdel/13ntdel)^* or *Pklr^(246ntdel/246ntdel)^*] specific for the RBC pyruvate kinase isoform (PKR) differentially affects blood cell counts in AA, AS, and SS Townes mice. Data are shown as scatter dot plots illustrating individual mouse measurements, with overlaid bars representing the least-squares mean ± 95% CI. Data were analyzed using a 2-way ANOVA, and *P* values were adjusted for multiple comparisons using the Tukey method. All experimental groups included balanced number of age- and sex-matched mice. As expected, SS*Pklr^(WT/WT)^* mice had leukocytosis (**A**) and anemia shown by lower RBC (**D**), hemoglobin (**G**), and hematocrit (**J**) compared with AA*Pklr^(WT/WT)^* and AS*Pklr^(WT/WT)^*. PKR-deficient [*Pklr^(13ntdel/13ntdel)^* and *Pklr^(246ntdel/246ntdel)^*] AA, AS, or SS mice had similar WBC counts compared with their respective *Pklr^(WT/WT)^* counterparts, **A**–**C**. In AA, AS, and SS mice, *Pklr* null mutations [*Pklr^(13ntdel/13ntdel)^* or *Pklr^(246ntdel/246ntdel)^*] yielded no significant changes in WBC (**A**–**C**). However, AA and AS mice with *Pklr* null mutations [AA*Pklr^(13ntdel/13ntdel)^*, AA*Pklr^(246ntdel/246ntdel)^*, AS*Pklr^(13ntdel/13ntdel)^*, AS*Pklr^(246ntdel/246ntdel)^*] developed anemia as shown by lower RBC counts (**D**–**F**), hemoglobin (**G**–**I**), and hematocrit (**J**–**L**) compared with AA*Pklr^(WT/WT)^* and AS*Pklr^(WT/WT)^*. In contrast, PKR-deficient SS mice [SS*Pklr^(13ntdel/13ntdel)^* or SS*Pklr^(246ntdel/246ntdel)^*] had similar RBC, hemoglobin, and hematocrit compared with SS*Pklr^(WT/WT)^* (**D**–**L**).

**Figure 3:**
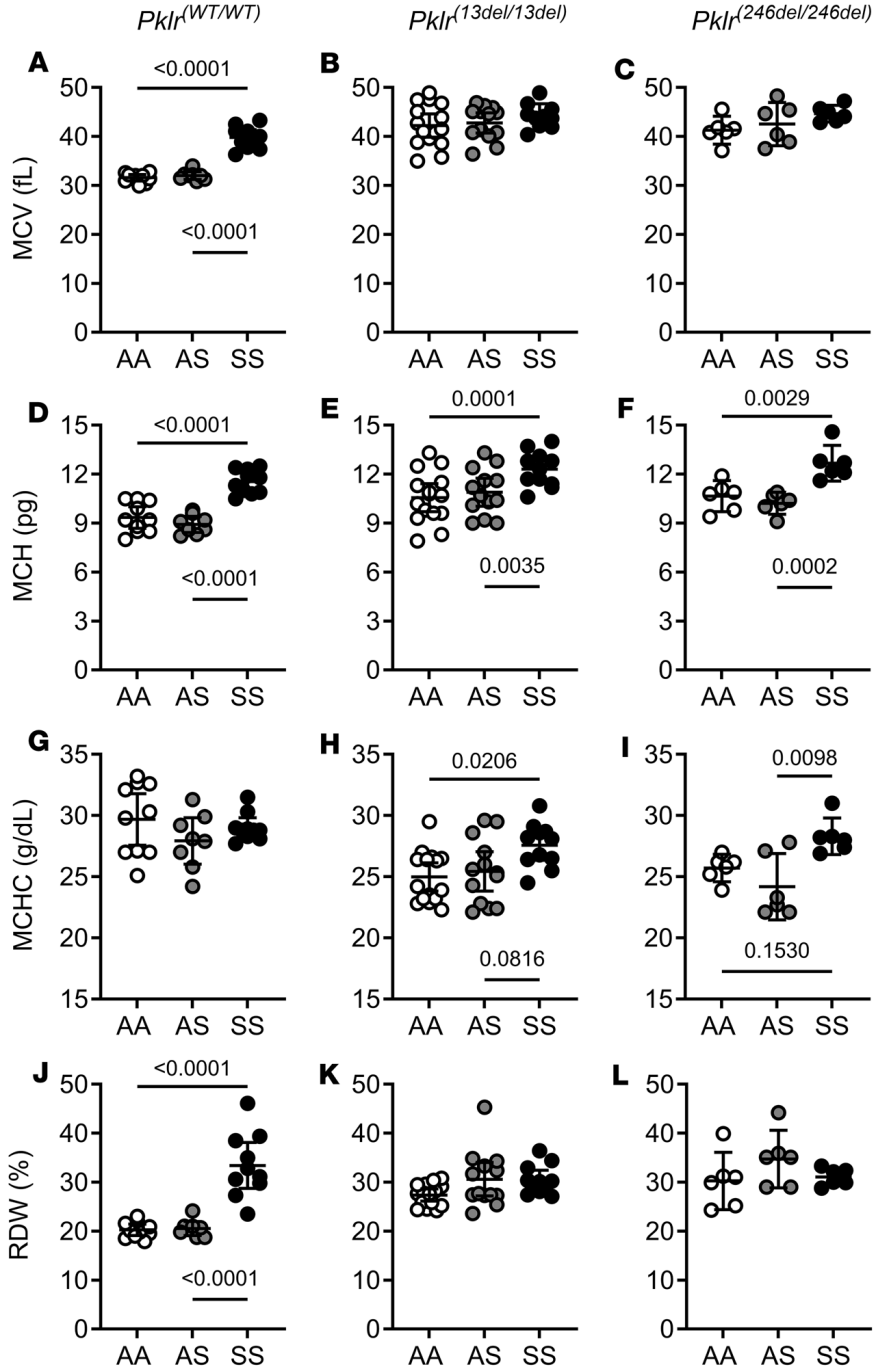
Coinheritance of *Pklr*-null mutations [*Pklr^(13ntdel/13ntdel)^* or *Pklr^(246ntdel/246ntdel)^*] specific for the RBC pyruvate kinase isoform (PKR) differentially affects hematologic indices in AA, AS, and SS Townes mice. Data are shown as scatter dot plots illustrating individual mouse measurements, with overlaid bars representing the least-squares mean ± 95% CI. Data were analyzed using a 2-way ANOVA, and *P* values were adjusted for multiple comparisons using the Tukey method. All experimental groups included balanced number of age- and sex-matched mice. Among animals with *Pklr^(WT/WT)^*, SS*Pklr^(WT/WT)^* mice had higher mean corpuscular volume (MCV, **A**), mean corpuscular hemoglobin (MCH, **D**), and red blood cell distribution width (RDW, **J**) (all *P* < 0.0001) and similar MCHC (**G**) compared with AA*Pklr^(WT/WT)^* and AS*Pklr^(WT/WT)^*. AA and AS mice with *Pklr-*null mutations [AA*Pklr^(13ntdel/13ntdel)^*, AA*Pklr^(246ntdel/246ntdel)^*, AS*Pklr^(13ntdel/13ntdel)^*, or AS*Pklr^(246ntdel/246ntdel)^*] had higher MCV, MCH, and RDW and lower mean corpuscular hemoglobin concentration (MCHC) compared with AA*Pklr^(WT/WT)^* and AS*Pklr^(WT/WT)^* (**A**, **B**, **D**, **E**, **G**, **H**, **J**, and **K**). In contrast, SS*Pklr^(13ntdel/13ntdel)^* and SS*Pklr^(246ntdel/246ntdel)^*) had higher MCV (**C**) but similar MCH (**F**), MCHC (**I**), and RDW (**L**) compared with SS*Pklr^(WT/WT)^*.

We next investigated the effect of PKR deficiency on hematologic parameters (Figures 2 and 3, and Supplemental Table 1). Coinheritance of PKR deficiency did not significantly affect WBC, RBC, hemoglobin or hematocrit in SS mice, as those outcomes were similar in SS*Pklr^(WT/WT)^* and PKR-deficient SS mice [*Pklr^(13ntdel/13ntdel)^* and *Pklr^(246ntdel/246ntdel)^*] (Figure 2, A–L, and Supplemental Table 1). In contrast, PKR-deficient AA and AS mice [*Pklr^(13ntdel/13ntdel)^* and *Pklr^(246ntdel/246ntdel)^*] developed severe anemia shown by lower RBC, hemoglobin and hematocrit compared with AA*Pklr^(WT/WT)^* and AS*Pklr^(WT/WT)^*, respectively (Figure 2, D–L, and Supplemental Table 1). Both AA and AS mice were similarly affected by PKR deficiency.

PKR deficiency minimally affected hematologic indices in SS mice. While PKR-deficient SS mice had further elevations in mean corpuscular volume (MCV, *P* < 0.005; Figure 3, A–C, and Supplemental Table 1), they exhibited similar mean corpuscular hemoglobin (MCH), mean corpuscular hemoglobin concentration (MCHC), and RBC distribution width (RDW) compared with SS*Pklr^(WT/WT)^* (Figure 3, D–L, and Supplemental Table 1). Conversely, PKR deficiency changed all hematological indices in AA and AS mice. Both PKR-deficient AA and AS were similarly affected, with elevated MCV, MCH, and RDW and lower MCHC compared with AA*Pklr^(WT/WT)^* and AS*Pklr^(WT/WT)^*, respectively (Figure 3, A–L, and Supplemental Table 1). These findings indicate that PKR deficiency differentially influenced hematologic parameters in AA and AS compared with SS mice (sickle genotype-by-*Pklr* mutation interactions, *P* ≤ 0.0217 for blood counts and hematological indices).

Lastly, PKR-deficiency yielded no significant changes in WBC (Figure 2, A–C, and Supplemental Table 1) or platelet counts (data not shown) in AA, AS, and SS mice.

### Coinheritance of PKR deficiency [Pklr^(13ntdel/13ntdel)^ or Pklr^(246ntdel/246ntdel)^] modulates sickling kinetics in AS and SS mice.

**Figure 4:**
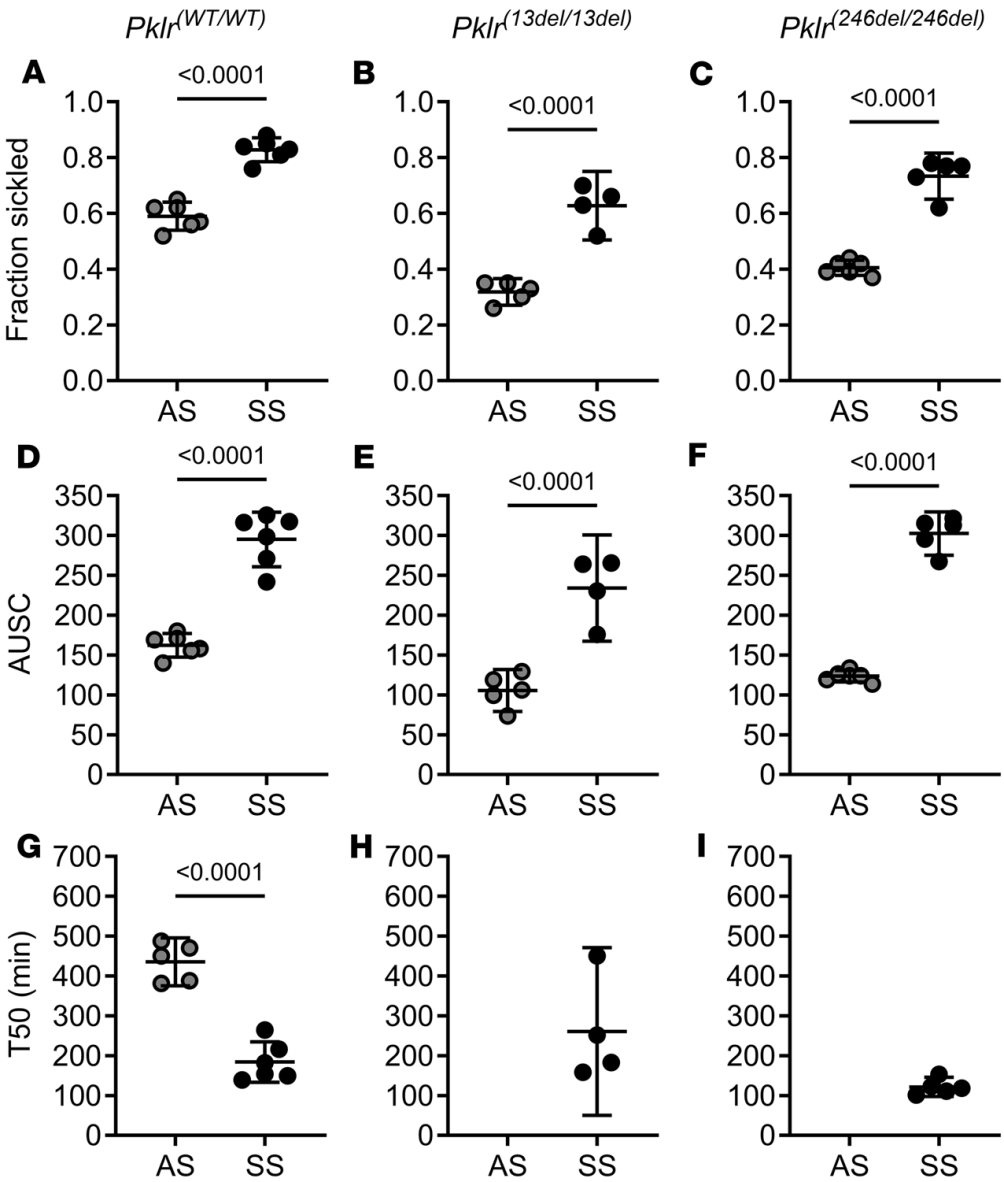
Coinheritance of *Pklr*-null mutations [*Pklr^(13ntdel/13ntdel)^* or *Pklr^(246ntdel/246ntdel)^*] specific for the RBC pyruvate kinase isoform (PKR) affects sickling kinetics in AS and SS mice. Data are shown as scatter dot plots illustrating individual mouse measurements, with overlaid bars representing the least-squares mean ± 95% CI. Data were analyzed using a 2-way ANOVA, and *P* values were adjusted for multiple comparisons using the Tukey method. All experimental groups included a balanced number of age- and sex-matched mice. When deoxygenated, RBCs from SS*Pklr^(WT/WT)^* mice had a higher percentage of sickled RBC (**A**), larger area under the sickling curve (AUSC, **D**), and a shorter T50 (time when 50% of RBCs are sickled, **G**) compared with AS*Pklr^(WT/WT)^*. Surprisingly, coinheritance of *Pklr^(13ntdel/13ntdel)^* or *Pklr^(246ntdel/246ntdel)^* in AS and SS mice decreased sickling as indicated by a decrease in the fraction of sickled RBC compared with AS*Pklr^(WT/WT)^* and *Pklr^(WT/WT)^* (**A**–**C**). Additionally, AS*Pklr^(13ntdel/13ntdel)^* and SS*Pklr^(13ntdel/13ntdel)^*, but not AS*Pklr^(246ntdel/246ntdel)^* or SS*Pklr^(246ntdel/246ntdel)^*, had a lower AUSC compared with AS*Pklr^(WT/WT)^* and SS*Pklr^(WT/WT)^* (**D**–**F**). While PKR deficiency [*Pklr^(246ntdel/246ntdel)^* or *Pklr^(246ntdel/246ntdel)^*] did not affect T50 in SS mice, RBCs from AS*Pklr^(13ntdel/13ntdel)^* and AS*Pklr^(246ntdel/246ntdel)^* did not reach T50 during the time limit of the assay, suggesting a prolongation of T50 (**G**–**I**)

When deoxygenated, RBCs containing HbS form polymers leading to sickling. As expected, RBCs from SS*Pklr^(WT/WT)^* mice had a higher percentage of sickled RBCs, larger area under the sickling curve (AUSC), and a shorter T50 (time in which 50% of RBCs are sickled) compared with AS*Pklr^(WT/WT)^* (all *P* < 0.0001, Figure 4, A, D, and G). Surprisingly, PKR deficiency reduced sickling both in AS and SS mice as shown by a lower fraction of sickled RBCs compared with their WT counterparts AS*Pklr^(WT/WT)^* and SS*Pklr^(WT/WT)^* (all *P* ≤ 0.0338; Figure 4, A–C, and Supplemental Table 1). The effect on the AUSC was specific to the *Pklr* mutation, in that PKR-deficient AS and SS mice carrying *Pklr^(13ntdel/13ntdel)^* exhibited a smaller AUSC compared with AS*Pklr^(WT/WT)^* and SS*Pklr^(WT/WT)^*, respectively (*P* = 0.0389 and *P* = 0.0359; Figure 4, D–F, and Supplemental Table 1). Conversely, no significant changes in AUSC were observed in AS or SS mice carrying the *Pklr^(246ntdel/246ntdel)^* mutation. Lastly, while PKR deficiency did not affect T50 in SS mice, AS*Pklr^(13ntdel/13ntdel)^* and AS*Pklr^(246ntdel/246ntdel)^* did not reach T50, indicating that less than 50% of the RBCs sickled by the end of the sickling assay time (Figure 4, G–I, and Supplemental Table 1).

### Coinheritance of PKR deficiency [Pklr^(13ntdel/13ntdel)^ or Pklr^(246ntdel/246ntdel)^] enhances extramedullary hematopoiesis in AA, AS, and SS mice.

**Figure 5:**
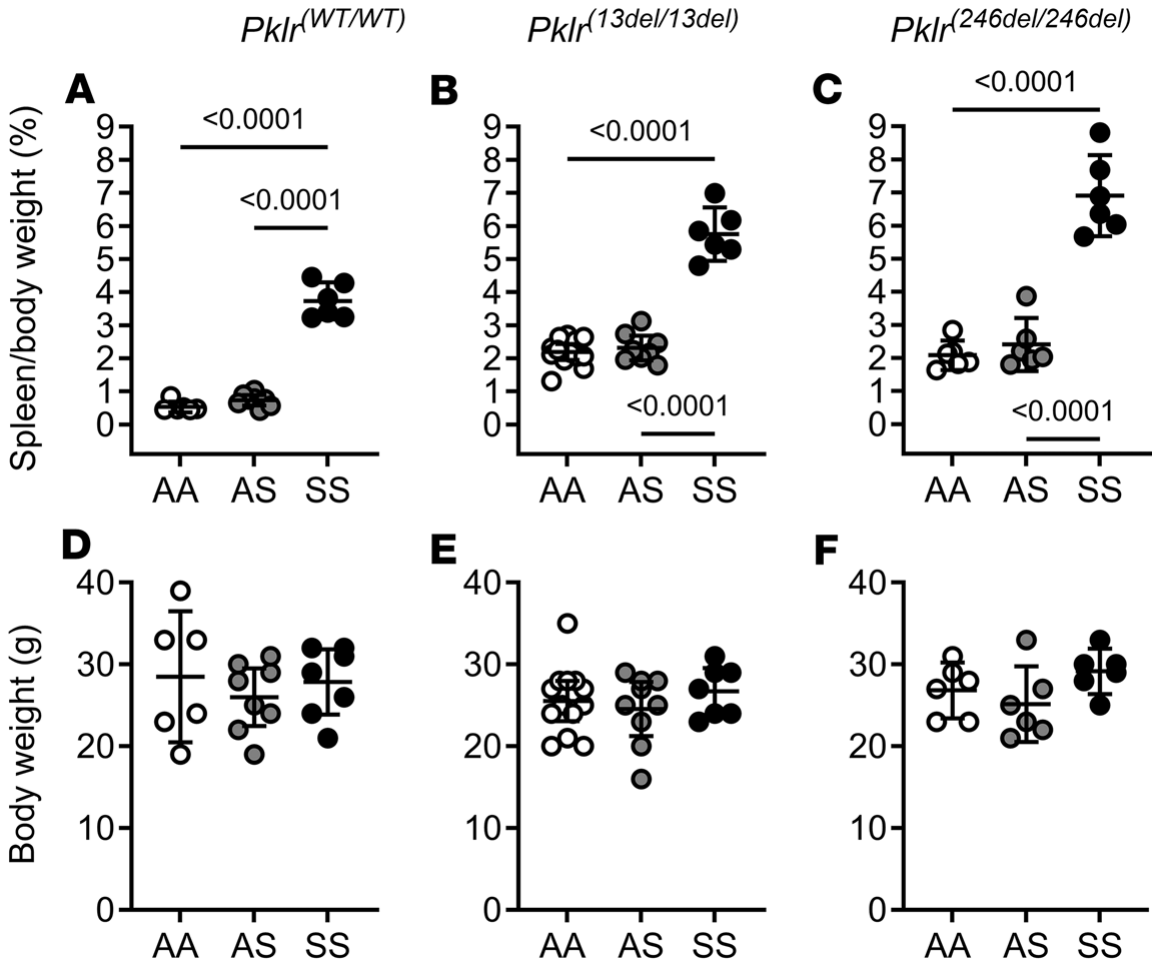
Coinheritance of *Pklr*-null mutations [*Pklr^(13ntdel/13ntdel)^* or *Pklr^(246ntdel/246ntdel)^*] specific for the RBC pyruvate kinase isoform (PKR) enhances extramedullary hematopoiesis in AA, AS, and SS mice. Data are shown as scatter dot plots illustrating individual mouse measurements, with overlaid bars representing the least-squares mean ± 95% CI. Data were analyzed using a 2-way ANOVA, and *P* values were adjusted for multiple comparisons using the Tukey method. All experimental groups included a balanced number of age- and sex-matched mice. SS*Pklr^(WT/WT)^* mice had higher spleen to body weight ratio compared with AA*Pklr^(WT/WT)^* and AS*Pklr^(WT/WT^*, (both *P* < 0.0001; **A**). PKR deficiency in AA, AS, SS mice was associated with an increase in spleen/body weight ratio compared with their respective *Pklr^(WT/WT)^* (all *P* < 0.0001; **A**–**C**) indicating a significant increase in extramedullary hematopoiesis. Of note, AA, AS, or SS mice with PKR deficiency had no significant changes in body weight (**D**–**F**).

As previously reported (26), SS*Pklr^(WT/WT)^* mice display enhanced extramedullary hematopoiesis as shown by increased spleen/body weight ratio compared with AA*Pklr^(WT/WT)^* and AS*Pklr^(WT/WT)^* (*P* < 0.0001; Figure 5, A–C, and Supplemental Table 1). PKR deficiency was associated with increased extramedullary hematopoiesis in all genotypes. PKR-deficient AA, AS, and SS mice had increased spleen/body weight ratio compared with their respective *Pklr^(WT/WT)^* counterparts (all *P* < 0.0001; Figure 5, A–C).

Regardless of genotype, males were heavier than female mice (*P* < 0.001, data not shown), and overall, PKR-deficient AA, AS, or SS mice had no significant changes in body weight (Figure 5, D–F).

### Coinheritance of PKR deficiency [Pklr^(13ntdel/13ntdel)^] affects mitochondrial content and function in circulating RBCs from AA, AS, and SS mice.

**Figure 6:**
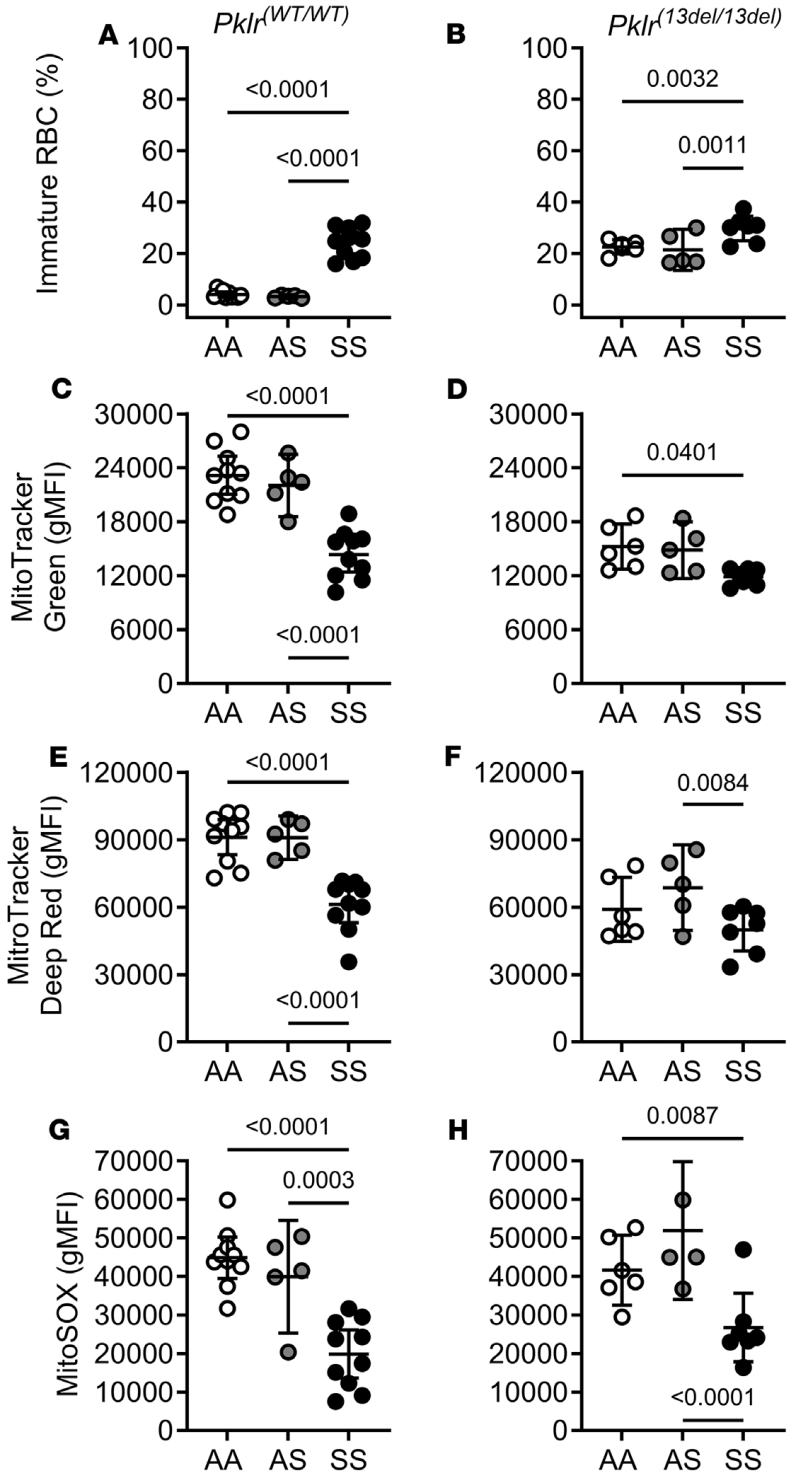
Coinheritance of *Pklr*-null mutation [*Pklr^(13ntdel/13ntdel)^*] specific for the RBC pyruvate kinase isoform (PKR) affects mitochondrial content and function in circulating immature RBCs from AA, AS, and SS mice. Data are shown as scatter dot plots illustrating individual mouse measurements, with overlaid bars representing the least-squares mean ± 95% CI. Data were analyzed using a 2-way ANOVA, and *P* values were adjusted for multiple comparisons using the Tukey method. All experimental groups included a balanced number of age- and sex-matched mice. Flow cytometric analysis of mitochondria in circulating immature RBCs (CD71^hi^ expression; **A** and **B**), which are predominantly reticulocytes, was carried out using MitoTracker Green (geometric mean fluorescence intensity, gMFI; **C** and **D**), MitoTracker Deep Red (**E** and **F**), and MitoSOX probes (**G** and **H**), which reflect mitochondrial mass, membrane potential, and superoxide content respectively. (**A**) SS*Pklr^(WT/WT)^* mice had a higher percentage of circulating immature RBCs (reticulocytes, approximately 24%) compared with AA*Pklr^(WT/WT)^* (4%) and AS*Pklr^(WT/WT)^* (3%). Immature RBCs from SS*Pklr^(WT/WT)^* displayed reduced mitochondrial mass (**C**), membrane potential (**E**), and superoxide content (**G**) compared with AA*Pklr^(WT/WT)^* and AS*Pklr^(WT/WT)^*. PKR-deficient [*Pklr*^(13ntdel/13ntdel)^] AA, AS, and SS mice had a higher percentage of immature RBCs compared with their respective *Pklr^(WT/WT)^* counterpart (*P* < 0.0001, *P* < 0.0001, and *P* = 0.0099; **A** and **B**). Immature RBCs from PKR-deficient AA and AS mice had reduced mitochondrial mass (**C** and **D**) and mitochondrial polarization (**E** and **F**) (all *P* ≤ 0.0034) but similar superoxide content (**G** and **H**) compared with AA*Pklr^(WT/WT)^* and AS*Pklr^(WT/WT)^*. In contrast, immature RBCs, from PKR-deficient SS had no significant changes in mitochondrial content or function compared with SS*Pklr^(WT/WT)^* mice (**C**–**H**).

**Figure 7:**
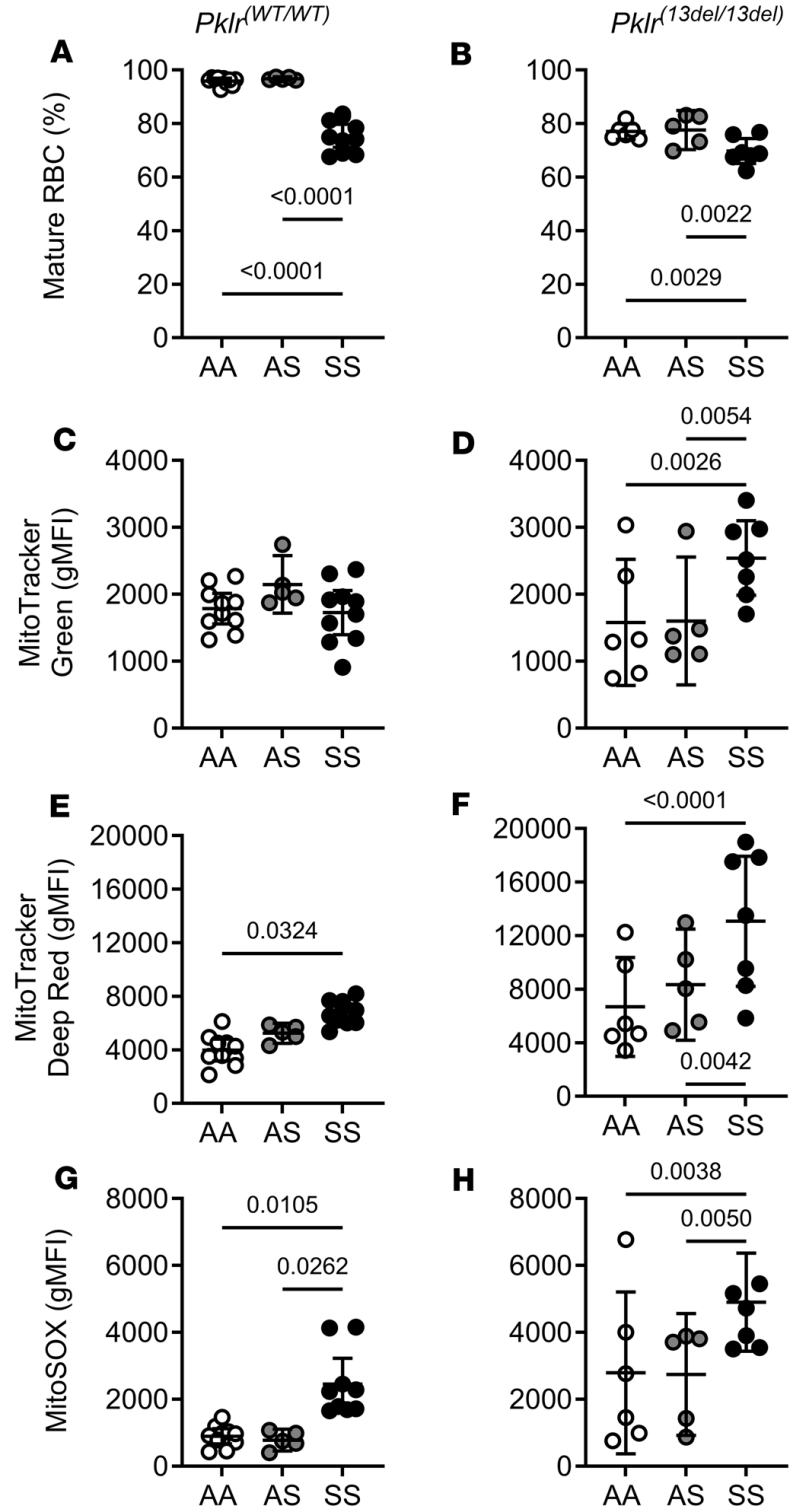
Coinheritance of *Pklr*-null mutation [*Pklr^(13ntdel/13ntdel)^*] specific for the RBC pyruvate kinase isoform (PKR) affects mitochondrial content and function in circulating mature RBCs from AA, AS, and SS mice. Data are shown as scatter dot plots illustrating individual mouse measurements, with overlaid bars representing the least-squares mean ± 95% CI. Data were analyzed using a 2-way ANOVA, and *P* values were adjusted for multiple comparisons using the Tukey method. All experimental groups included balanced number of age- and sex-matched mice. Flow cytometric analysis of mitochondria in circulating mature RBCs (CD71^low^ expression) was carried out using MitoTracker Green (geometric mean fluorescence intensity, gMFI; **C** and **D**) and MitoTracker Deep Red (**E** and **F**), and MitoSOX probes (**G** and **H**), which reflect mitochondrial mass, membrane potential, and superoxide content respectively. SS*Pklr^(WT/WT)^* mice had a lower percentage of circulating mature RBCs (**A**), which contained higher degree of polarized mitochondria (**E**), and superoxide content (**G**) compared with AA*Pklr^(WT/WT)^*. PKR deficient AA, AS, and SS mice had lower percentage of circulating mature RBCs compared with their respective *Pklr^(WT/WT)^* counterparts, (*P* < 0.0001, *P* < 0.0001, and *P* = 0.0101; **A** and **B**). RBCs from PKR-deficient AA mice exhibited elevated mitochondrial membrane potential (*P* = 0.0143; **E** and **F**), and superoxide content (*P* = 0.0054; **G** and **H**) compared with AA*Pklr*^(WT/WT)^, suggesting that functional mitochondria were retained in those mature RBCs. PKR-deficient AS had higher RBC mitochondrial superoxide content (*P* = 0.0212; **G** and **H**) compared with AS*Pklr^(WT/WT)^*. Lastly, mature RBCs from PKR-deficient SS mice had elevated mitochondrial mass (*P* = 0.0041; **C** and **D**) and further elevations in mitochondrial polarization (*P* = 0.0007; **E** and **F**) and superoxide content (*P* = 0.0002; **G** and **H**) compared with SS*Pklr^(WT/WT)^*.

Using flow cytometry and mitochondrial markers, we examined mitochondrial mass (MitoTracker Green), membrane potential (MitoTracker Deep Red), and superoxide (MitoSOX) content in immature (TER119^+^ and CD71^hi^, which are predominantly reticulocytes) and mature (TER119^+^ and CD71^low^) RBCs from AA, AS, and SS mice with and without PKR deficiency (Figure 6, Figure 7, and Supplemental Figure 3). As previously shown (26, 27), SS*Pklr^(WT/WT)^* mice have a higher percentage of circulating immature RBCs (predominantly reticulocytes, 24%) compared with AA*Pklr^(WT/WT)^* (4%) and AS*Pklr^(WT/WT)^* (3%), (all *P* < 0.0001; Figure 6A, and Supplemental Table 2).

PKR-deficient [*Pklr*^(13ntdel/13ntdel)^] AA, AS, and SS mice showed an elevated percentage of immature RBCs compared with their respective *Pklr^(WT/WT)^* counterpart (*P* < 0.0001, *P* < 0.0001 and *P* = 0.0099, respectively; Figure 6, A and B, and Supplemental Table 2). Immature RBCs from PKR-deficient AA and AS mice showed reduced mitochondrial mass and mitochondrial polarization (all *P* ≤ 0.0034) but had similar superoxide content compared with AA*Pklr^(WT/WT)^* and AS*Pklr^(WT/WT)^* (Figure 6, C–H, Supplemental Figure 3, and Supplemental Table 2). In contrast, immature RBCs from PKR-deficient SS mice showed no significant changes in mitochondrial content or function compared with SS*Pklr^(WT/WT)^* mice (Figure 6, C–H, Supplemental Figure 3, and Supplemental Table 2).

As we had previously shown (26, 28) mature RBCs from SS*Pklr^(WT/WT)^* contained a higher degree of polarized mitochondria (*P* = 0.0324) and superoxide content (*P* = 0.0105) compared with AA*Pklr^(WT/WT)^* (Figure 7, A, E, and G, and Supplemental Table 2), indicating mitochondrial retention.

PKR deficiency was associated with a decrease in mature RBCs. PKR-deficient AA, AS, and SS mice had a lower percentage of mature RBCs compared with their respective *Pklr^(WT/WT)^* counterparts (*P* < 0.0001, *P* < 0.0001 and *P* = 0.0101, respectively; Figure 7, A–C, and Supplemental Table 2). PKR-deficient AA mice exhibited elevated mitochondrial membrane potential (*P* = 0.0143; Figure 7, E and F; Supplemental Figure 3; and Supplemental Table 2), and superoxide content (*P* = 0.0054; Figure 7, G and H, and Supplemental Figure 3) compared with AA*Pklr*^(WT/WT)^, indicating that mitochondria were retained in those mature RBCs. PKR-deficient AS had higher RBC mitochondrial superoxide content (*P* = 0.0212; Figure 7, G and H, Supplemental Figure 3, and Supplemental Table 2) compared with AS*Pklr^(WT/WT)^*. Lastly, mature RBCs from PKR-deficient SS mice had elevated mass and further elevations in mitochondrial polarization and superoxide content (*P* = 0.0041, *P* = 0.0007, and *P* = 0.0002, respectively; Figure 7, C–H, Supplemental Figure 3, and Supplemental Table 2) compared with SS*Pklr^(WT/WT)^*.

Using whole blood transmission electron microscopy (TEM) images (8 images per each mouse), we determined the average number of mitochondria per mature RBC (Supplemental Figure 4) (24, 29–31). While RBCs from SS*Pklr^(WT/WT)^* mice exhibited a higher average number of mitochondria per RBC compared with AA*Pklr^(WT/WT)^* (*P* = 0.0084) and AS*Pklr^(WT/WT)^* (*P* = 0.0128; Supplemental Figure 4A), PKR deficiency independently increased RBC mitochondrial retention in all genotypes. PKR-deficient AA, AS, and SS mice had a higher average number of mitochondria per RBC compared with their respective *Pklr^(WT/WT)^* counterpart (*P* = 0.0010, *P* = 0.0016, and *P* = 0.0378, respectively; Supplemental Figure 4, A–H, and Supplemental Table 2).

### Coinheritance of PKR deficiency differentially affects liver and spleen histopathology in AA, AS, and SS mice.

**Figure 8:**
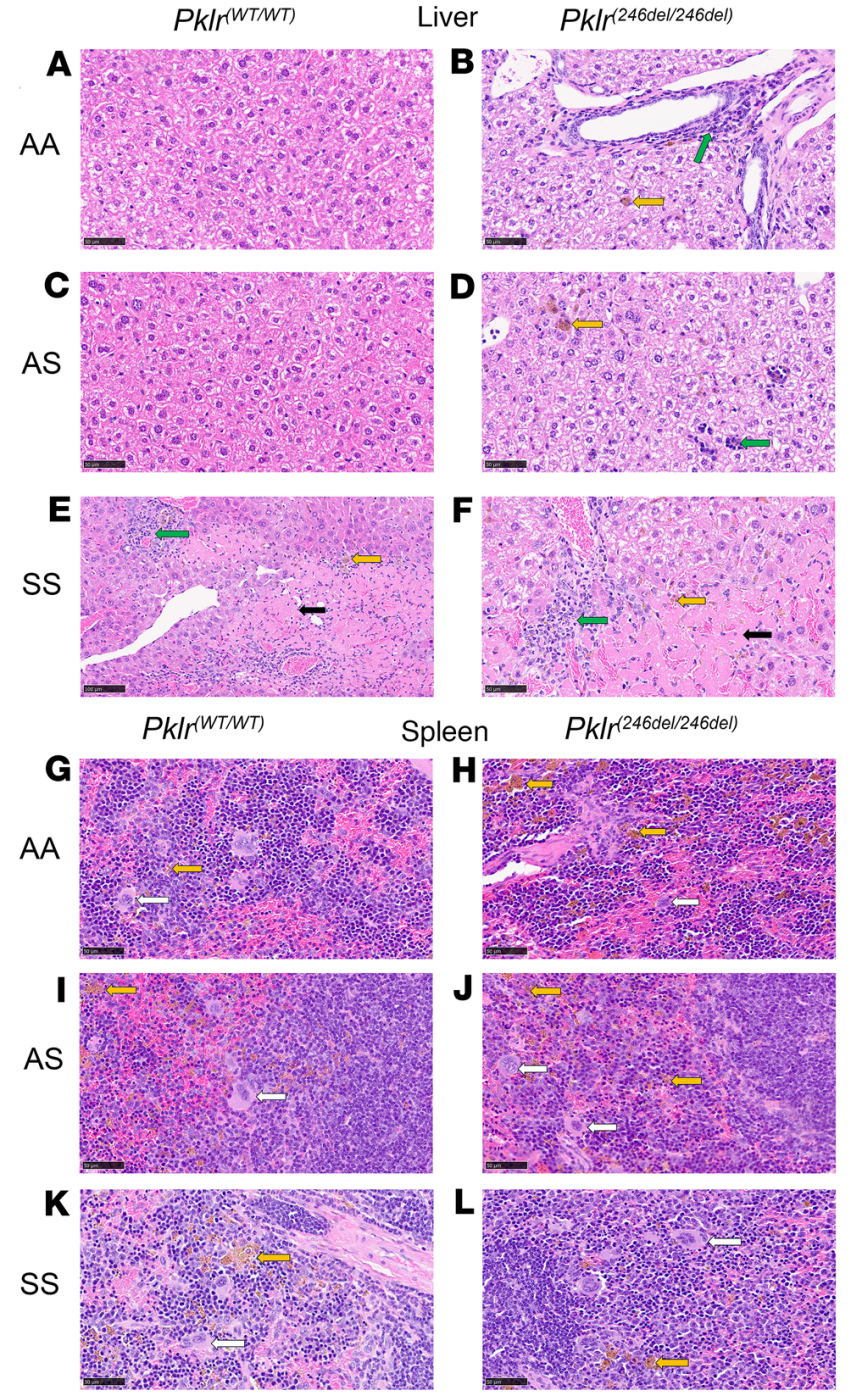
Coinheritance of *Pklr*-null mutations [*Pklr^(13ntdel/13ntdel)^* or *Pklr^(246ntdel/246ntdel)^*] specific for the RBC pyruvate kinase isoform (PKR) differentially affects liver and spleen histopathology in AA, AS, and SS mice. Each panel shows representative H&E-stained sections of liver and spleen from *Pklr^(WT/WT)^*and *Pklr^(246ntdel/246ntdel)^*. Yellow arrows indicate hemosiderin pigmentation in macrophages, green arrows lymphocytic infiltration (inflammation), black arrow necrosis, and white arrows extramedullary hematopoiesis. Mice with *Pklr^(13ntdel/13ntdel)^* and *Pklr^(246ntdel/246ntdel)^* had similar histopathology; for simplicity, we only display representative images for *Pklr^(246ntdel/246ntdel)^* mice. Compared with AA*Pklr^(WT/WT)^* (**A**) and AS*Pklr^(WT/WT)^* (**C**), and SS*Pklr^(WT/WT)^* (**E**) mice displayed marked hepatic inflammation (lymphocytes and macrophage infiltration), necrosis, and iron deposition (hemosiderin pigmentation in macrophages). PKR-deficient AA (**B** and **H**) and AS mice (**D** and **J**) developed substantial liver and spleen iron deposition, as well as marked hepatic inflammation, and increased extramedullary hematopoiesis compared with AA*Pklr^(WT/WT)^* (**A** and **G**) or AS*Pklr^(WT/WT)^* (**C** and **I**). In contrast, PKR-deficient SS (**F** and **L**) showed no significant changes in liver or spleen histopathology compared with SS*Pklr^(WT/WT^* mice (**E** and **K**). Liver and spleen histopathology from 5 to 12 mice per each genotype were examined.

As previously reported SS*Pklr^(WT/WT)^* mice display marked hepatic inflammation (lymphocytes and macrophage infiltration), necrosis, and iron deposition (hemosiderin pigmentation in macrophages) (32), findings which were not present in AA*Pklr^(WT/WT)^* or AS*Pklr^(WT/WT)^* (Figure 8). PKR-deficient AA and AS mice [*Pklr ^(13ntdel/13ntdel)^* or *Pklr*^(246ntdel/246ntdel)^] developed substantial spleen and liver iron deposits, as well as marked hepatic inflammation, and increased extramedullary hematopoiesis compared with AA*Pklr^(WT/WT)^* or AS*Pklr^(WT/WT)^* (Figure 8). In contrast, PKR-deficient SS mice showed no significant changes in liver or spleen histopathology compared with SS*Pklr*^(WT/WT)^ (Figure 8). PKR-deficient AA, AS, and SS mice displayed no significant changes on kidney pathology (data not shown).

### Coinheritance of PKR deficiency mildly affects biochemical parameters in AA, AS, and SS mice.

Overall, PKR-deficient AA, AS, and SS, mice had higher total bilirubin (*P* < 0.0001, for overall *Pklr* mutation effect) and blood urea nitrogen (BUN, *P* = 0.0299) compared with *Pklr^(WT/WT)^* counterparts (Supplemental Figure 5, A–F, and Supplemental Table 2). Lastly, AA*Pklr^(13ntdel/13ntdel)^* had higher protein (*P* = 0.0063) and globulin (*P* = 0.0122) levels compared with AA*Pklr^(WT/WT)^* (Supplemental Figures 5, G, I, J, and L, and Supplemental Table 2).

### Coinheritance of PKR deficiency differentially affects grip force in AA, AS, and SS mice.

Overall, controlling for *Pklr* mutations and sickle genotype, females had higher grip force compared with males (*P* < 0.0001; Supplemental Figure 6). Among females, SS*Pklr^(WT/WT)^* and AS*Pklr^(WT/WT)^* mice had lower grip force in the forelimbs and all limbs compared with AA *Pklr^(WT/WT)^* (Supplemental Figure 6, A and D). PKR-deficient AA, but not AS or SS females, had lower all-limbs grip force compared with AA*Pklr^(WT/WT)^* (*P* = 0.0015; Supplemental Figure 6, D and F, and Supplemental Table 2).

### Coinheritance of Pklr^(WT/13ntdel)^ or Pklr^(WT/246ntdel)^ did not alter the phenotype of AA, AS, and SS mice.

AA, AS, and SS mice with coinheritance of *Pklr^(WT/13ntdel)^* or *Pklr^(WT/246ntdel)^* mutations displayed similar ATP and 2,3-DPG levels, hematological and biochemical parameters, sickling kinetics, spleen and body weight, grip force profile, and mitochondrial content and function in mature and immature RBCs compared with *Pklr^(WT/WT)^* controls (Supplemental Figures 5–13).

## Discussion

We confirmed that coinheritance of PKR deficiency modifies the phenotypes of Townes AA (β^A^/β^A^, controls), AS (β^A^/β^S^, HbS carrier), and SS (β^S^/β^S^, SCD) mice. PKR-deficient AA mice exhibited hematologic and pathologic features consistent with those observed in humans with PK deficiency (PKD) (33–35). PKR-deficient AS mice mirrored the clinical presentation of individuals with SCT who coinherit PKD and develop SCD (17, 18). Both PKR-deficient AA and AS mice developed severe anemia, marked reticulocytosis, mitochondrial retention in mature RBCs, increased extramedullary hematopoiesis, splenic iron deposition, and hepatic inflammation with iron accumulation. The anemia in PKR-deficient AA and AS mice was associated with elevated MCV and RDW and decreased MCHC. These findings likely reflect increased reticulocytosis; however, given the concomitant increase in ATP levels in those animals, the possibility of increased RBC hydration cannot be excluded. In contrast, PKR-deficient SS mice exhibited surprisingly mild phenotypic changes. Compared with SS*Pklr^(WT/WT)^*, PKR-deficient SS mice showed further increases in reticulocytosis, enhanced extramedullary hematopoiesis, and increased mitochondrial retention in mature RBCs but displayed no significant changes in anemia severity or organ pathology. Notably, despite developing increases in 2,3-DPG levels, PKR-deficient SS mice displayed decreased sickling, a finding also observed in PKR-deficient AS, which did not have increased 2,3-DPG. Collectively, these findings support the notion that *Pklr*-null mutations can profoundly modify the hematologic and organ injury phenotypes in HbS carrier models and, to a lesser extent, in SCD mouse models.

We found that PKR-deficient mice [*Pklr^(13ntdel/13ntdel)^* or *Pklr*^(246ntdel/246ntdel)^] with AA and AS genotypes had higher blood ATP levels compared with their *Pklr^(WT/WT)^* counterparts. While PKR-deficient SS mice also had high blood ATP, their levels were similar to the already-elevated levels found in SS*Pklr^(WT/WT)^*. These results were unexpected because a metabolic block at the PKR-dependent ATP-generating step of glycolysis is expected to reduce pyruvate and ATP and to increase upstream glycolytic metabolites, including 2,3-DPG. Interestingly, these findings are consistent with metabolomic analysis of RBCs from patients with PKD revealing elevated ATP levels despite decreased pyruvate and lactate (36, 37). These increases in ATP could reflect compensatory mechanisms, such as reduced ATP consumption or heightened activity at upstream glycolysis pathway steps as suggested by others (36, 37). Another possibility is that ATP was produced by oxidative phosphorylation in reticulocytes or in mature RBCs that have retained mitochondria in PKR-deficient AA, AS, and SS mice. Supporting this hypothesis are findings from human PKD RBCs, which have elevated levels of TCA cycle metabolites, increased oxygen consumption, and a reduction in RBC ATP content when oxidative phosphorylation is inhibited by cyanide or hypoxia (36, 37). Our earlier data further support this hypothesis, showing that mitochondrial inhibitors decrease ATP levels by 30%–60% in RBCs from SS mice (38). Ultimately, these results suggest that PKR deficiency leads to significant reprogramming of RBC metabolism and bioenergetics in AA and AS mice, a phenomenon that was less pronounced in SS mice.

Unlike humans with SCD, SS*Pklr^(WT/WT)^* exhibit increased baseline RBC PK activity, reflected by higher ATP and lower 2,3-DPG blood levels compared with control mice (24, 39). We found that PKR-deficient SS mice had elevated 2,3-DPG without significant changes in ATP levels compared with SS*Pklr^(WT/WT)^*. Given the increases in 2,3-DPG, we hypothesized that PKR-deficient SS mice would have increased sickling as 2,3-DPG stabilizes the sickle fiber, thus promoting sickling (15). Contrary to our hypothesis, PKR-deficient SS mice displayed decreased sickling, an effect also observed in PKR-deficient AS, even though these animals had no changes in 2,3-DPG. In PKR-deficient AS mice, decreased sickling could be explained by the increases in ATP, which can improve RBC hydration (40), membrane integrity, and deformability (41–43), as well as by the decreases in MCHC, which lowers HbS concentration and increases delay time to polymerization (15). However, the mechanism underlying decreased sickling in PKR-deficient SS is unclear. Although these mice exhibited additional increases in MCV, which could reflect increased RBC hydration, their MCHC and ATP levels were unchanged. While beyond the scope of this work, investigation into the metabolic and biophysical consequences of PKR deficiency in SS RBCs will add insights into the effect of PKR on RBC metabolism and RBC sickling.

We and others have shown that mature RBCs from humans and mice with SCD abnormally retain mitochondria (24, 28, 44). Here we found that PKR-deficient SS mice [SS*Pklr^(13ntdel/13ntdel)^*] displayed further increases in mitochondrial retention as evidenced by an elevated mitochondrial mass, increased membrane polarization, and heightened superoxide production in mature RBCs compared with SS*Pklr^(WT/WT)^* (Figure 7 and Supplemental Figures 3 and 4). Furthermore, PKR-deficient AA and AS mice developed anemia and retained mitochondria as well as had increased superoxide levels in mature RBCs. Since the final stages of erythroid cell maturation involve the removal of mitochondria and other organelles from immature RBCs as they transition into fully mature cells (45), these findings suggest that PKR deficiency might have affected erythroid maturation in AA, AS, and SS mice. Supporting this hypothesis are several studies showing that patients and animals with PKD have altered maturation of erythroid progenitors and disrupted levels of key regulators of iron balance and erythropoiesis, leading to ineffective erythropoiesis (46, 47). In hematologic conditions such as β-thalassemia (48) and SCD (28), the RBC maturation process is disrupted and mature RBCs retain mitochondria, which reflects ineffective erythropoiesis and contribute to the pathobiology of these diseases. Further support for the role of PK activity in erythropoiesis comes from findings in mouse models of β-thalassemia and SCD, where treatment with a PK activator (mitapivat) increases blood ATP levels, reduces mitochondrial retention, and decreases the abundance of mitochondrial proteins in RBCs, collectively suggesting an enhancement in effective erythropoiesis and improvement in erythroid maturation (24, 49). Given the critical interplay between mitochondrial metabolism and glycolysis during erythroid maturation (45, 50), our findings support the possibility that glycolysis disruption due to PKR deficiency may alter the metabolic programing of erythroid precursors, disrupt erythroid maturation, and lead to increased mitochondrial retention in mature RBCs in AA, AS, and SS mice.

In humans, PKD is characterized by hemolysis, anemia, reticulocytosis, extramedullary hematopoiesis, and iron overload (33–35). PKR-deficient AA mice recapitulate these phenotypes and also exhibit elevated blood ATP levels, a finding previously reported in both patients with PKD (36, 37) and PKD murine models (51). While canine (52, 53) and murine models of PKD have been described (51, 54–56), our AA PKR-deficient model presents distinct advantages. Specifically, the AA*Pklr^(13ntdel/13ntdel)^* and AA*Pklr^(246ntdel/246ntdel)^* strains express human HbA and were engineered on a different genetic background strain with unique *Pklr* mutations (13 or 246 nucleotide deletions) unlike those in earlier mouse models (54–56). Despite these differences, AA*Pklr^(13ntdel/13ntdel)^* and AA*Pklr^(246ntdel/246ntdel)^* mice display phenotypes consistent with those described in existing murine models of PKD and in the human disease. Collectively, these findings indicate that mouse models of PKD replicate the genotype/phenotype heterogeneity observed in patients with PKD. The AA*Pklr^(13ntdel/13ntdel)^* and AA*Pklr^(246ntdel/246ntdel)^* mice thus expand the existing preclinical models and represent valuable tools for mechanistic studies and therapeutic development in PKD.

In patients with SCD, *PKLR* variants are linked to increased hospitalizations for sickle cell pain (16). To investigate the potential effect of PKR deficiency on pain-related phenotypes, we examined grip strength, a surrogate measure of muscle pain, in AA, AS, and SS mice. SS mice exhibit decreased grip strength compared with AA and AS, a finding believed to reflect muscle hyperalgesia (57–59). SS*Pklr^(13ntdel/13ntdel)^* exhibited no significant changes in grip strength, whereas AA*Pklr^(13ntdel/13ntdel)^* mice developed decreases in grip strength. Possibly, AA*Pklr^(13ntdel/13ntdel)^* had decreases in grip strength because of anemia. The lack of change in grip strength in SS*Pklr^(13ntdel/13ntdel)^* mice might be explained by a possible increase in muscle oxygen delivery due to increases in 2,3-DPG. While we did not explore other pain behaviors in AA and SS mice, our findings indicate that PKR deficiency does not affect muscle hyperalgesia in SS mice.

In summary, our findings demonstrate that *Pklr-*null mutations in AA mice accurately mirror the hematologic and histopathologic hallmarks of human PKD, thereby validating this model as a robust experimental system for the disorder. We also showed that PKR deficiency modulates the phenotypes of HbS carriers, significantly affecting AS mice, and alters specific parameters in SS mice. Although the phenotypes observed in PKR -deficient SS mice do not fully mirror those observed in patients, this discrepancy likely reflects intrinsic differences in the glycolytic metabolism between murine models with SCD and humans. Nevertheless, PKR -deficient SS mice underscore the complexity of genotype/phenotype relationships and demonstrate that PKR deficiency serves as a key modifier of HbS-associated pathology, even in the setting of advanced disease. Considering the observed alterations in RBC metabolites and mitochondrial retention, these models provide a valuable tool for investigations of the complex relationships among glycolysis, RBC metabolism and bioenergetics, and erythroid maturation, thereby advancing our understanding of how PKR activity modulates the pathophysiology of SCT and SCD.

## Methods

Supplemental Methods are available online with this article.

### Sex as a biological variable.

In this study, we included a balanced number of age-matched male and female animals in all experimental groups. While we did not power the study to examine the effects of sex, we included this variable in the analyses and report the results for both sexes except when there was a sex effect, such as on body weight and grip force.

### Generation of Pklr mutations in Townes sickle cell mice and study design.

We used the B6;129 Hba^tm1(HBA)Tow^Hbb^tm2(HBG1,HBB*)Tow^/*Hbb^tm3(HBG1,HBB)Tow^*/J strain (Strain #:013071, The Jackson Laboratory), here referred to as the Townes model (26, 60, 61). In the model, mouse hemoglobin genes were knocked out and human hemoglobin genes were knocked in. AA mice express human HbA, AS human HbA and HbS, and SS mice human HbS (26, 59–62). Sickle genotyping was performed as previously described (59, 62). We utilized CRISPR-Cas9 gene editing technology (63) to generate targeted loss-of-function mutations in the *Pkl*r gene in Townes AS mouse embryos. Five mutant alleles were generated, screened, and 2 null mutant alleles were selected: 13 nt and 246 nt deletions in the promoter of P*klr* that is specific for PKR (Supplemental Figures 1 and 2). Mice with coinheritance of selected *Pklr* mutations were generated and maintained in AA, AS, and SS Townes mice, and the mice included in this study were backcrossed for at least 5 generations.

### Outcome measurements of blood analysis.

We collected blood from anesthetized animals and measured complete blood counts [Element HT5 (Heska Corporation)] and plasma biochemistry [Element DC5X (Heska Corporation)]. Whole blood ATP and 2,3-DPG levels were measured by Agios Pharmaceuticals using liquid chromatography–tandem mass spectrometry (LC-MS/MS) and normalized to hematocrit as described (24).

The fraction of sickled RBCs as a function of time was measured by deoxygenating RBCs with nitrogen using a Biotek Lionheart FX automated microscope system and gas controller (Agilent Technologies) in a 37°C humidified chamber. The degree of sickling was determined from the fraction sickled at the end of the ~500-minute assay, the time at which 50% of the cells sickled (T50) and the area under the sickling curve (AUSC) (64).

Mitochondrial retention in mature (TER119^+^ and CD71^lo^) and immature RBCs, which are predominantly reticulocytes (TER119^+^ and CD71^hi^) was examined by flow cytometry (CytoFlex Analyzer, Beckman Coulter) using the following markers: Calcein-FITC (Invitrogen #C3100MP), MitoTracker Green-FITC (MTG, Invitrogen #M7514), MitoSOX-PE (Invitrogen #M36008) and MitoTracker Deep Red-APC (MTDR, Invitrogen #M22426), TER119-PECy7 (Invitrogen #25-5921-81), CD71-AlexaFluor700 (Invitrogen #56-0711-82), CD45-APCeFluor780 (Invitrogen #47-0451-82), and CD41a-APCeFluor780 (Invitrogen #47-0411-82). We also used TEM to quantify the average number of mitochondria in RBCs as previously described (24, 29–31).

### Organ histopathology.

After blood collection, animals were euthanized, and liver, spleen, and kidneys were collected and fixed in 10% buffered formalin for histopathological evaluation.

### Grip strength.

We measured mouse grip strength using the Grip Strength Meter (GSM, San Diego Inc.) as described (58, 59).

### Statistics.

For each outcome (dependent) variable, a 3-way ANOVA model was fitted with the following explanatory (independent) variables: mouse sickle genotype, *Pklr* mutation, sex, and the following interaction terms: sickle genotype-by-*Pklr* mutation, sickle genotype-by-sex, *Pklr* mutation-by-sex, and sickle genotype-by-*Pklr* mutation-by-sex. When the 3-way interaction (sickle genotype × *Pklr* mutation × sex) was not significant, sex was removed from the model and a 2-way ANOVA including sickle genotype, *Pklr* mutation, and their interaction was fitted. Least-squares means and corresponding 95% CI were obtained from the models. Model fit diagnostics were examined to determine whether model assumptions were met. In the results, when reporting overall genotype effects, those were controlled for *Pklr* mutation and sex. Similarly, overall effects of *Pklr* mutation were controlled for sickle genotype and sex. Differences between groups were interpreted and stated based on *P* values adjusted for post-hoc pairwise comparisons using the Tukey method. *P* < 0.05 was considered statistically significant.

### Study approval.

The NIH Clinical Center Animal Care and Use Committee approved all animal procedures (DPM 23-01, DPM23-02, DPM23-03). All experimental procedures complied with the *Guide for the Care and Use of Laboratory Animals* (National Academies Press, 2011).

### Data availability.

All data needed to evaluate the conclusions are presented the manuscript, its figures, and in the Supporting Data Values file. Any additional information required to reanalyze the data reported in this paper is available upon request to the corresponding author.

## Author contributions

Concept and design of the study were contributed by WAE, SLT, and ZMNQ. Data acquisition was contributed by XW, MS, SK, SV, LEFA, QL, MBT, KYS, NS, MQ, and CL. Analyzes and interpretation of data were contributed by RV, XW, WAE, SLT, ZMNQ, and CL. MS and ZMNQ wrote the first draft. All authors reviewed, edited, and approved the manuscript. XW and MS are co–first authors and contributed equally; authorship order assigned by flipping a coin. SLT and ZMNQ are co–senior authors and contributed equally; authorship order assigned by flipping a coin.

## Funding support

This work is the result of NIH funding, in whole or in part, and is subject to the NIH Public Access Policy. Through acceptance of this federal funding, the NIH has been given a right to make the work publicly available in PubMed Central.

- Intramural Research Program of the NIH Clinical Center
- National Heart Lung and Blood Institute
- National Institute of Diabetes and Digestive and Kidney Diseases
- National Cancer Institute
- National Eye Institute, NIH

## Supplementary Material

Supplemental data

Unedited blot and gel images

Supporting data values

## Version 1 01/20/2026

In-Press Preview

## Version 2 02/23/2026

Electronic publication
