# Supplementary material for: Pyruvate kinase deficiency modifies sickle hemoglobin carrier and sickle cell disease phenotypes in mice
Source: JCI Insight. 2026 Jan 8;11(4):e195682. doi: 10.1172/jci.insight.195682 (PMC12956008; doi:10.1172/jci.insight.195682)
Supplement: Supplemental data [file jciinsight-11-195682-s057.pdf]

Pyruvate kinase deficiency modifies sickle hemoglobin carrier and sickle cell disease phenotypes in mice

Xunde Wang[1*], Meghann L. Smith[2*], Sayuri Kamimura[2], Quan Li[3], Niharika Shah[4], Martha Quezado[4], Luis E.F. Almeida[2], Sebastian Vogel[2], Mickias Tegegn[1], Kevin Y. Sun[1], Rafael Villasmil[5], Chengyu Liu[6], William A. Eaton[3], Swee Lay Thein[1†], and Zenaide M.N. Quezado[1,2†]

*Co-first authors

†Co-senior authors

[1]Sickle Cell Branch, National Heart Lung and Blood Institute, National Institutes of Health, Bethesda, MD 20892, USA
[2]Department of Perioperative Medicine, National Institutes of Health Clinical Center, National Institutes of Health, Bethesda, MD 20892, USA
[3]Laboratory of Chemical Physics, National Institute of Diabetes and Digestive and Kidney Diseases, National Institutes of Health, Bethesda, MD 20892, USA
[4]Laboratory of Pathology, National Cancer Institute, National Institutes of Health, Bethesda, MD 20892, USA
[5]Flow Cytometry Core Facility, National Eye Institute, National Institutes of Health, Bethesda, MD
[6]National Heart Lung and Blood Institute, National Institutes of Health, Bethesda, MD 20892, USA

Correspondence: Zenaide (Zena) M.N. Quezado, MD
Department of Perioperative Medicine
NIH Clinical Center
National Institutes of Health
Bethesda, MD 20892
E-mail: zquezado@nih.gov

Swee Lay Thein, MD
Sickle Cell Branch, National Heart Lung and Blood Institute,
National Institutes of Health, Bethesda, MD 20892, USA
E-mail: sweelay.thein@nih.gov

Supplemental Methods

Generation of *Pklr* mutations on the Townes mouse model of sickle cell disease

The $Pklr^{(13ntdel/13ntdel)}$ and $Pklr^{(246ntdel/246ntdel)}$ mutations were generated using the CRISPR/Cas9 method as previously described (1). Briefly, a single guide RNA (sgRNA, TAACTGCTGGTCT**TATC**TGC, with the four bold nucleotides representing the GATA motif in reverse orientation) was designed to cut in the *Pklr* gene promoter between the CTCTG and GATA motifs. These motifs are required for driving the expression of the erythroid-specific pyruvate kinase (PKR) isoform but are not required for expression of PK isoforms found in other tissues. The sgRNA was synthesized in vitro using ThermoFisher's sgRNA In Vitro Transcription Service. The sgRNA (20ng/µL) was co-microinjected with Cas9 mRNA (50 ng/µL), TriLink BioTechnologies) into the cytoplasm of zygotes collected from Townes sickle cell disease (SCD) mouse mating pairs. Injected embryos were cultured overnight in KSOM medium (Millipore Sigma) in a 37ºC incubator with 6% CO_2. In the next morning, embryos which reached the 2-cell stage of development were implanted into the oviducts of pseudo pregnant surrogate mothers (CD1 mice from Charles River Laboratory). Offspring born to the foster mothers were genotyped using PCR (Forward primer: 5'-GGATTGTATCACTGGCGATG-3'; Reverse primer: 5'-CTGGCACAGACGAGATCAGT-3') followed by Sanger sequencing. One founder mouse with a 13bp deletion and another founder mouse with a 246bp deletion around the sgRNA cutting site were expanded by breeding with Townes SCD mice to establish the two mutant strains described in this study.

AS mice heterozygous for the 13nt or 246nt *Pklr* deletions were intercrossed to establish a colony of Townes AA (β^A/β^A, controls), AS (β^A/β^S, sickle hemoglobin carrier), and SS (β^S/β^S,

sickle cell disease) with and without co-inheritance of *Pklr* mutations. We then examined the genotype/phenotype relationships in AA, AS, and SS animals with co-inheritance of *Pklr*$^{(WT/13ntdel)}$, *Pklr*$^{(13ntdel/13ntdel)}$, *Pklr*$^{(WT/246ntdel)}$, *or Pklr*$^{(246ntdel/246ntdel)}$ mutations.

In all experiments and experimental groups, we included balanced numbers of age-matched (16 to 30 weeks) male and female mice. Blood was collected from animals anesthetized with isoflurane using cardiac puncture and heparinized syringes. Following anesthesia and exsanguination, liver, spleen, and kidneys were collected and fixed in 10% buffered formalin for histological evaluation with hematoxylin and eosin stains. Analysis of blood and tissue samples were performed by investigators unaware of the animals' genotypes.

Immunoblot assay for pyruvate kinase (PK) PKR and PKM isoforms

Mouse RBCs were washed three times with PBS. Lysates were obtained by adding radioimmunoprecipitation assay (RIPA) buffer, supplemented with a protease inhibitor cocktail (Complete Tablets, Roche, Indianapolis, IN), followed by sonication. The lysates were centrifuged at 10,000 × g at 4C for 5 minutes. The protein concentration of the supernatant was determined, and the lysates were mixed with 4X NuPAGE LDS sample buffer for subsequent immunoblotting. Total protein of 20 µg was loaded onto a 4-12% SDS NuPAGE gel and transferred to a 0.45 µm nitrocellulose membrane. Anti-PKR antibody (Invitrogen:PA5-34734, Carlsbad, CA) and anti-PKM antibody (Cell signaling, cat # 3190, Danvers, MA) were used for detection.

Liver tissues were collected, frozen in dry-ice alcohol, and stored at -80°C. Lysates were prepared by homogenizing tissue in RIPA buffer, followed by centrifugation at 10,000 × g for 10

minutes. The supernatant was collected, and protein concentrations were determined. SDS-PAGE and immunoblotting were performed as described for the red blood cell lysates.

Complete blood counts and plasma biochemistry

We measured WBC and RBC counts, platelets, hemoglobin, hematocrit, mean corpuscular volume (MCV), mean corpuscular hemoglobin (MCH), mean corpuscular hemoglobin concentration (MCHC), and red cell distribution width (RDW) using the Element HT5 (Heska Corporation, Loveland, CO). We then separated plasma by centrifugation and measured electrolytes, blood urea nitrogen (BUN), creatinine, phosphorus, calcium, total protein, albumin, globulin, cholesterol, alanine aminotransferase, Aspartate aminotransferase, and total bilirubin using the Element DC5X (Heska Corporation, Loveland, CO).

Whole blood adenosine triphosphate (ATP) and 2,3-diphosphoglycerate (2,3-DPG) quantification

Whole blood levels of ATP and 2,3-DPG were determined utilizing previously described (2, 3) liquid chromatography tandem mass spectrometry (LC-MS/MS) method. Briefly, whole blood (5 μL) was mixed with water (10 μL) containing the internal standards $^{13}C_3$-2,3-DPG (200 μg/mL) and $^{13}C_{10}$,$^{15}N_5$-ATP (200 μg/mL). Samples were vortex-mixed and centrifuged at 3000 rpm for one minute. 40 μL of water was added, plate vortex-mixed for approximately 10 minutes and centrifuged for another minute. Samples were then extracted with 320 μL of methanol, mixed for 10 min and then centrifuged at 3000 rpm for 10 minutes. A 40 μL supernatant was transferred to another plate and 320 μL of acetonitrile was added. The mixture

was mixed for 2 minutes, followed by centrifugation at 4000 rpm for 5 minutes. The resulting supernatant was transferred to an injection plate for LC-MS/MS analysis. An ion exchange method was used for separation of ATP and 2,3 DPG on a BioBasic AX analytical column (2.1 X 50 mm, 5 µM, Thermo Fisher Scientific, Waltham, MA). Detection was done using a SCIEX Triple Quad 6500 mass spectrometer (SCIEX. Framingham, MA) in negative electrospray ionization mode. Calibration curves were established using ATP and 2,3 DPG authentic standards (50 to 2000 µg/mL). The peak area ratios of the analyte relative to the internal standard were used for quantitation and whole blood levels of ATP and 2,3-DPG were normalized to sample hematocrit.

Sickling assay

Additional details for the sickling assay have been previously reported (4, 5). When HbS polymerizes upon deoxygenation in RBCs, the cell shape is distorted (6), which is readily detected using an optical microscope (4, 5). Images of AS and SS erythrocytes suspended in a pH 7.3 300 mOsM phosphate buffered saline solution (32 mM Na_2HPO_4, 8 mM KH_2PO_4, 130 mM NaCl, 1 mg/mL BSA, 1 mg/mL dextrose) were collected following the initiation of deoxygenation using a Lionheart FX automated microscope system (Agilent technologies) in a 37°C humidified and atmosphere-controlled chamber; nitrogen flow was regulated to reach and maintain 5% oxygen for SS samples and 0% for AS samples inside the instrument. Deoxygenation of the cells requires about 30 min. Several metrics were used to determine the time at which a cell sickles, loss of circular shape, loss of more transparent center characteristic of a biconcave disc, and a decrease in area of the cell. The output of the assay is the fraction of sickled RBCs as a function of time after the initiation of deoxygenation, from which the time

required for 50% of the cells to sickle (t50) is obtained. An increase in t50 corresponds to a decrease in sickling.

Flow cytometry and mitochondria staining of circulating RBCs

Whole blood was assayed by flow cytometry using CytoFlex (Beckman Coulter). Samples were labelled following a two-step staining protocol. First, aliquots of mouse whole blood with 10uM Calcein-FITC (Invitrogen #C3100MP, Carlsbad, CA), 100nM MitoTracker Green-FITC (Invitrogen #M7514), 5uM MitoSOX-PE (Invitrogen #M36008) and/or 100nM MitoTracker Deep Red-APC (Invitrogen #M22426) were incubated in Hank's balanced salt solution (HBSS) with Ca^{+2}/Mg^{+2} at 37°C for 30min (protected from the light), followed by centrifugation (1,000g at RT for 5min) and resuspension of cells in 500ul HBSS (with Ca^{+2}/Mg^{+2} supplemented with 2% FBS). Then, surface antibodies TER119-PECy7 (Invitrogen #25-5921-81), CD71-AlexaFluor700 (Invitrogen #56-0711-82), CD45-APCeFluor780 (Invitrogen #47-0451-82) and CD41a-APCeFluor780 (Invitrogen #47-0411-82) were added and incubated at RT for 25min (protected from the light), followed by centrifugation (1,000g at RT for 5 min) and resuspension of cells into 500ul HBSS (with Ca^{+2}/Mg^{+2} supplemented with 2% FBS) for acquisition.

Three aliquots from each mouse sample were prepared, one for each of the mitochondrial markers. Calcein-FITC was added to all aliquots, except to the one containing MitoTracker Green-FITC. Control mouse samples, single staining vials, FMOs and unstained samples were all included. Flow cytometry results were analyzed using FlowJo™ v10.10 Software (BD Life Sciences, Franklin Lakes, NJ) and FlowJo plugin flowAI (7). Labeling and gating strategies excluded dead cells, doublets, platelets and WBC. The same strategies were consistently applied

to all experiments and data was generated by plotting CD71 vs. TER119. Cells expressing higher levels of CD71 ($CD71^{high}$) and TER119 were considered immature RBCs, which in Townes mice are predominantly reticulocytes, and those expressing TER119 and low CD71 ($CD71^{low}$) were considered mature RBCs.

Whole blood transmission electron microscopy (TEM) and quantification of mitochondria in erythrocytes

We also evaluated RBC mitochondrial retention using whole blood TEM as previously described (2, 8, 9). Whole blood was fixed in 2.5% glutaraldehyde and 1% paraformaldehyde in 0.1M cacodylate buffer (pH 7.4) and washed with cacodylate buffer three times. The sample was centrifuged at 2400g for two minutes, fixed with 1% OsO_4, washed again with cacodylate buffer times, washed with water and placed in 1% uranyl acetate for 30 minutes. Subsequently, samples were serially dehydrated in ethanol and propylene oxide and embedded in EMBed 812 resin (Electron Microscopy Sciences, Hatfield, PA). Sections (approximately 80 nm) were obtained using the Leica ultracut-UCT ultramicrotome (Leica, Deerfield, IL) and placed onto 300 mesh copper grids and stained with UranyLess (Electron Microscopy Sciences, Hatfield, PA) and then with lead citrate. The grids were viewed with a JEM-1200EXII electron microscope (JEOL Ltd, Tokyo, Japan) at 80kV and images were recorded on the XR611M, mid mounted, 10.5M pixel, CCD camera (Advanced Microscopy Techniques Corp, Danvers, MA). Investigators, blinded to animal genotypes, analyzed eight whole blood TEM images per mouse. Within these images, individual, anuclear cells with a curved, elliptical shape were classified as mature RBCs. Intracellular organelles with a double-membrane and internal cristae were categorized as mitochondria. For each of the eight images per mouse, the number of

mitochondria within all visible mature RBC was quantified and the average number of mitochondria per RBC for each mouse was calculated (2, 10).

Organ histopathology

A pathologist blinded to the mouse experimental group evaluated H&E-stained spleen, liver, and kidney tissue sections for the presence of extramedullary hematopoiesis, necrosis, iron deposition, and inflammation. To quantify extracellular hematopoiesis and hemosiderosis, entire spleen sections were scanned under 200x magnification from at least 5 animals per experimental group. The presence of megakaryocytes was interpreted as to reflect extramedullary hematopoietic foci and of hemosiderin pigmentation in macrophages as iron deposition. We also examined lymphocytes, macrophages, or neutrophils tissue infiltrates, which were considered to reflect inflammation. Areas of necrosis in the liver were identified and evaluated based on presence of areas ranging from collection of individual cells with shrunken, more eosinophilic round body lacking a nucleus to cells with a small, shriveled nucleus, with or without accompanying inflammation to focal necrosis involving larger groups of hepatocytes within a lobule. The extent of inflammation, necrosis, and extramedullary hematopoietic were graded as follows 0: None, 1+: mild, 2+: moderate, 3+: severe

Grip strength

We measured forelimbs and all limbs (forelimbs and hind limbs together) grip strength using the Grip Strength Meter (GSM, San Diego, Inc., San Diego, CA) as described (11, 12). After training and acclimatization to the device, animals were held by the tail and allowed to grasp a steel grip gauge with forelimbs only or with all four limbs. Once animals griped the steel

grip gauge, they were gently and steadily pulled away until the grip was released. The force measured at time of grip release (either forelimbs only or all four limbs) was recorded as grip strength. We measured grip force daily for 4 days and analyzed the average grip force.

Supplemental Table 1. Outcome measurements in mice with co-inheritance of pyruvate kinase R isoform deficiency resulting from $Pklr^{(13ntdel/13ntdel)}$ or $Pklr^{(246ntdel/246ntdel)}$ null mutations in AA (β^A/β^A, control), AS (β^A/β^S, sickle hemoglobin carrier), and SS (β^S/β^S, sickle cell disease) mice compared to respective $Pklr^{(WT/WT)}$ counterparts

	$Pklr^{(WT/WT)}$ [A]			$Pklr^{(13ntdel/13ntdel)}$ [B]			$Pklr^{(246ntdel/246ntdel)}$ [C]		
**Variable**	**AA**	**AS**	**SS**	**AA**	**AS**	**SS**	**AA**	**AS**	**SS**
ATP (mM) [D]	0.86±0.17	0.81±0.11<br>p=0.9127	1.32±0.16<br>p= 0.0015	1.22±0.26<br>p=0.0112	1.24±0.12<br>p=0.0018	1.38±0.39<br>p=0.9689	1.46±.025<br>p=0.0006	1.26±0.45<br>p=0.0029	1.47±0.14<br>p=0.7318
2,3-DPG [E] (mM)	8.18±1.12	7.40±1.69<br>p=0.9127	4.61±0.47<br>p<0.0001	7.61±0.99<br>p=0.7991	7.28±0.57<br>p=0.9994	6.54±0.48<br>p=0.0145	7.26±0.10<br>p=0.6434	6.61±0.74<br>p=0.6275	6.76±1.15<br>p=0.0048
ATP/2,3-DPG ratio	0.11±0.01	0.11±0.02<br>p=0.9979	0.29±0.05<br>p<0.0001	0.16±0.03<br>p=0.0171	0.17±0.02<br>p=0.0134	0.21±0.05<br>p=0.0020	0.20±0.03<br>p=0.0007	0.19±0.05<br>p=0.0023	0.22±0.03<br>p=0.0123
WBC (10^9/L)	5.1±2.5	5.7±2.1<br>p=0.9767	21.3±12.5<br>p<0.0001	6.3±3.1<br>p=0.9870	6.7±2.6<br>p=0.9947	23.7±9.9<br>p=0.8928	6.3±3.6<br>p=0.9944	7.4±1.6<br>p=0.9820	22.3±9.9<br>p=0.9984
RBC (10^{12}/L)	8.59±1.05	8.58±1.21<br>p=0.9993	5.19±0.73<br>p<0.0001	5.19±0.73<br>p<0.0001	5.29±0.41<br>p<0.0001	4.94±0.74<br>p=0.9207	5.14±0.78<br>p<0.0001	4.90±0.86<br>p<0.0001	4.24±0.9<br>p=0.3981
Hemoglobin (g/dL)	8.39±0.90	7.69±1.35<br>P=0.3721	6.08±0.89<br>p<0.0001	5.49±1.07<br>p<0.0001	5.76±0.95<br>p=0.0006	6.05±0.78<br>p>0.9999	5.43±0.67<br>p<0.0001	4.98±0.71<br>p<0.0001	5.4±1.25<br>p=0.7119
Hematocrit (%)	28.4±1.9	27.4±3.8<br>P=0.8425	21.0±3.2<br>p<0.0001	22.0±3.9<br>p=0.0005	22.8±2.8<br>p=0.0253	22.0±2.8<br>p=0.9571	21.2±3.1<br>p=0.0015	20.9±4.0<br>p=0.0034	19.0±4.1<br>p=0.7012
MCV (fL)	31.6±0.9	32.0±0.9<br>p=0.9353	39.8±2.3<br>p<0.0001	42.2±4.3<br>p<0.0001	42.8±3.4<br>p<0.0001	44.7±3.0<br>p=0.0005	41.3±2.7<br>p<0.0001	42.5±4.2<br>p<0.0001	44.7±1.6<br>p=0.0050
MCH (pg)	9.4±0.9	8.9±0.6<br>p=0.6647	11.5±0.7<br>p<0.0001	10.6±1.6<br>p=0.0398	10.9±1.4<br>p=0.0005	12.3±1.1<br>p=0.9734	10.7±0.9<br>p=0.050	10.2±0.6<br>p=0.067	12.7±1.0<br>p=0.262
MCHC (g/dL)	29.7±2.9	27.9±2.3<br>p=0.2706	29.0±1.1<br>p=0.8068	25±2.1<br><0.0001	25.4±2.7<br>p=0.0670	27.6±1.8<br>p=0.6614	25.7±1.1<br>p=0.0144	24.2±2.6<br>p=0.0362	28.3±1.4<br>p=0.9992
RDW (%)	20.2±1.6	20.5±1.7<br>p=0.9864	33.4±6.6<br>p<0.0001	27.4±2.3<br>p=0.0002	30.6±5.7<br>p<0.0001	30.4± 3.0<br>p=0.9981	30.3±5.6<br>p<0.0001	34.7±5.6<br>p<0.0001	31.1±1.8<br>p=0.9891
Fraction sickled	–	0.59±0.05	0.83±0.04	–	0.32±0.04<br>p<0.0001	0.63±0.08<br>p<0.0001	–	0.41±0.03<br>p<0.0001	0.73±0.07<br>p=0.0338
AUSC	–	162±14	295±33	–	106±21<br>p=0.0389	234±42<br>p=0.0359	–	124±6.8<br>p=0.2334	303±22<br>p=0.9953
T50 (min)	–	436±48	184±48	–	↑	261±132<br>p=0.4619	–	↑	121±19<br>p=0.7146
Spleen/body weight (%)	0.53±0.16	0.73±0.19<br>p=0.7853	3.73±0.54<br>p<0.0001	2.19±0.40<br>p<0.0001	2.31±0.44<br>p<0.0001	5.76±0.77<br>p<0.0001	2.08±0.42<br>p<0.0001	2.40±0.76<br>p<0.0001	6.91±1.17<br>p<0.0001

Data are shown as mean ± SD. P values in **$Pklr^{(WT/WT)A}$** columns indicate comparisons between AS$Pklr^{(WT/WT)}$ or SS$Pklr^{(WT/WT)}$ with AA$Pklr^{(WT/WT)}$. P values in the **$Pklr^{(13ntdel/13ntdel)}$ [B]** and **$Pklr^{(246ntdel/246ntdel)}$ [C]** columns indicate comparisons between AA, AS, SS *Pklr* mutants [$Pklr^{(13ntdel/13ntdel)}$ or $Pklr^{(246ntdel/246ntdel)}$] and respective **$Pklr^{(WT/WT)}$** counterparts. ATP [D] and 2,3-diphosphoglycerate (2,3-DPG) [E] were normalized to hematocrit. MCV indicates mean corpuscular volume, MCH mean corpuscular hemoglobin, MCHC mean corpuscular hemoglobin concentration, RDW red cell distribution width, AUSC area under sickling curve, T50 time when 50% of RBC are sickled, ↑ increased, and – not performed.

Supplemental Table 2. Effects of co-inheritance of pyruvate kinase R isoform deficiency resulting from *Pklr*$^{(13ntdel/13ntdel)}$ null mutation in AA (β^A/β^A, control), AS (β^A/β^S, sickle hemoglobin carrier), and SS (β^S/β^S, sickle cell disease) mice compared to respective *Pklr*$^{(WT/WT)}$ counterparts

	***Pklr*$^{(WT/WT)}$ A**			***Pklr*$^{(13ntdel/13ntdel)}$ B**		
**Variable**	**AA**	**AS**	**SS**	**AA**	**AS**	**SS**
Immature RBC[C] (%) (Reticulocytes)	4.0±1.4	3.1±0.6 p=0.9133	24.1±5.9 p<0.0001	22.5±2.6 p<0.0001	21.4±6.4 p<0.0001	29.7±5.1 p=0.0099
Immature RBC (MTG, gMFI)	23160±2949	22036±2791 p=0.6729	14363±2720 p<0.0001	15239±2402 p<0.0001	14844±2547 p<0.0001	11882±918 p=0.1016
Immature RBC (MTDR, gMFI)	91132±10926	91004±7778 p=0.9997	61269±11424 p<0.0001	59053±13576 p<0.0001	68693±15390 p<0.0034	49914±10183 p=0.0747
Immature RBC (MitoSOX, gMFI)	44866±7484	39934±11779 p=0.5535	19863±8735 p<0.0001	41605±8652 p=0.7461	51928±14427 p=0.0812	26728±9624 p=0.2491
Mature RBC (%)	95.9±1.4	96.8±0.5 p=0.9040	75.4±6.0 p<0.0001	77.0±2.8 p<0.0001	77.6±5.9 p<0.0001	69.8±5.0 p=0.0101
Mature RBC (MTG, gMFI)	1785±320	2147±347 p=0.3787	1726±460 p=0.9614	1581±897 p=0.7012	1602±768 p=0.1959	2542±601 p=0.0041
Mature RBC (MTDR, gMFI)	3976±1137	5233±610 p=0.6102	6774±900 p=0.0324	6677±3516 p=0.0143	8336±3343 p=0.0918	13075±5261 p=0.0007
Mature RBC (MitoSOX, gMFI)	894±316	779±265 p=0.9807	2456±1001 p=0.0105	2790±2304 p=0.0054	2741±1468 p=0.0212	4906±1585 p=0.0002
Mitochondria/ RBC (average)	0.8±0.6	0.8±0.8 p>0.9999	2.3±0.7 p=0.0084	2.6±1.1 p=0.0010	2.5±0.5 p=0.0016	3.4±0.6 p=0.0378
Total bilirubin (mg/dL)	0.44±0.34	0.11±0.05 p=0.1555	0.7±0.31 p<0.001	0.48±0.18 p=0.9711	0.38±0.16 p=0.2444	1.16±0.48 p=0.0307
Blood urea nitrogen (mg/dL)	17.3±3.6	18.8±1.6 p=0.7982	19.8±4.4 p=0.5639	25.6±3.9 p=0.0041	26.5±2.9 p=0.0060	24.7±4.8 p=0.1246
Total protein (g/dL)	4.3±0.5	4.5±0.3 p=0.4209	4.7±0.2 p=0.0806	4.9±0.2 p=0.0063	4.6±0.3 p=0.7965	4.9±0.5 p=0.6111
Globulin (mg/dL)	2.3±0.3	2.4±0.2 p=0.3349	2.6±0.2 p=0.0122	2.6±0.2 p=0.0122	2.5±0.2 p=0.8723	2.8±0.3 p=0.8628
All limbs grip force females[D]	11.5±1.4	7.5±0.8 p=<0.0001	8.7±1.0 p=0.0009	8.8±1.0 p=0.0015	8.9±1.5 p=0.0720	8.3±0.9 p=0.6094

Data are shown as mean ± SD. ***Pklr*$^{(WT/WT)}$ A** columns show p values reflecting comparisons between AS*Pklr*$^{(WT/WT)}$ or SS*Pklr*$^{(WT/WT)}$ with AA*Pklr*$^{(WT/WT)}$. ***Pklr*$^{(13ntdel/13ntdel)}$ B** columns show p values reflecting comparisons between AA, AS, SS with co-inheritance of a *Pklr* null mutation [*Pklr*$^{(13ntdel/13ntdel)}$] and respective wild-type counterpart (*Pklr*$^{(WT/WT)}$). Immature RBCs [C] represent cells stained positive for TER119 with high expression of CD71, which in AA, AS, and SS mice are predominantly reticulocytes, whereas mature RBCs are TER119 positive cells with low CD71 expression. MTG indicates MitoTracker Green, gMFI geometric mean fluorescence intensity, and MTDR, MitoTracker Deep Red. The unit of measurement for grip force females [D] is g force/g of body weight.

Supplemental Figure 1

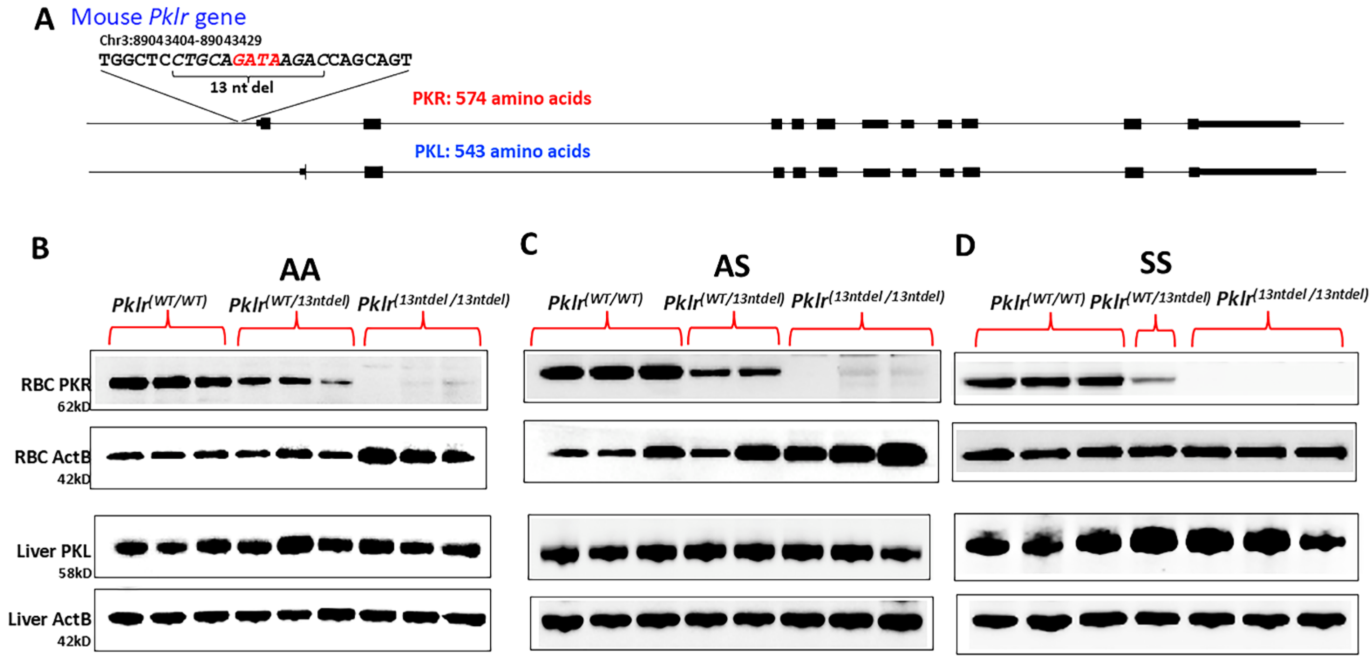

Supplemental Figure 1. AA, AS, and SS mice with co-inheritance of *Pklr* null mutations lack expression of RBC pyruvate kinase (PKR) isoform and have intact expression of the liver isoform PKL. (**A**) Schematic structure of the mouse *Pklr* gene and target of the 13nt deletion. **B**, **C**, and **D**. Immunoblot analysis of AA, AS, and SS mice with co-inheritance of $Pklr^{(WT/13ntdel)}$, $Pklr^{(13ntdel/13ntdel)}$. AA, AS, and SS mice $Pklr^{(13ntdel/13ntdel)}$ mutations have no detectable PKR protein in RBCs but intact expression of PKL in the liver confirming the specificity of the *Pklr* mutations for PKR. Similar results were observed in mice with $Pklr^{(246ntdel/246ntdel)}$ mutation (data not shown).

Supplemental Figure 2

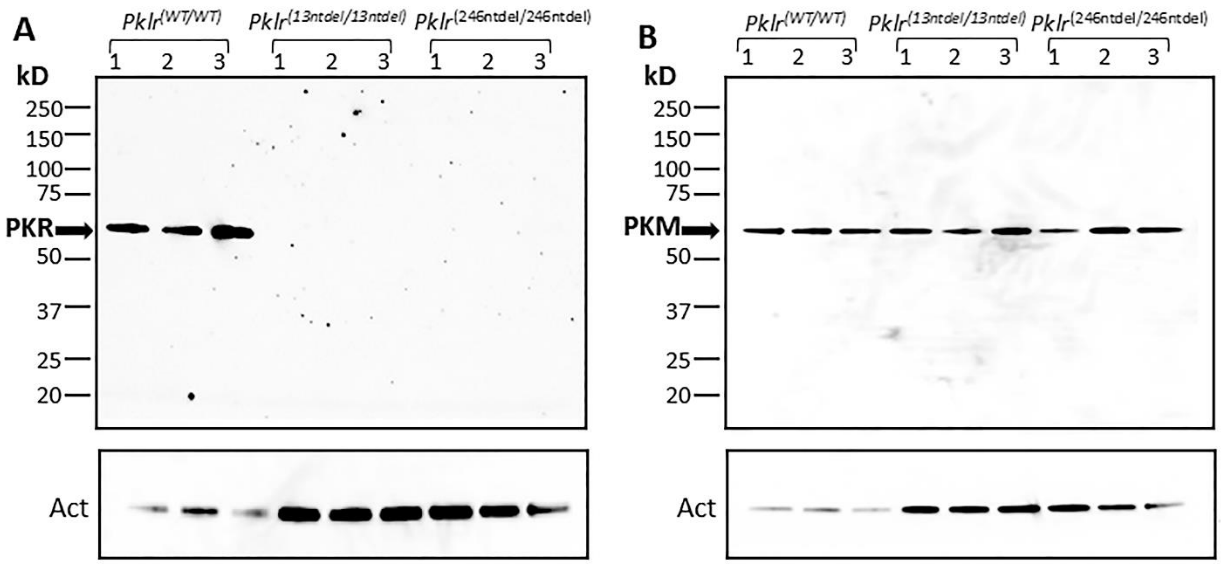

Supplemental Figure 2. AA, AS, and SS mice with co-inheritance of *Pklr* null mutations [*Pklr*$^{(13ntdel/13ntdel)}$ or *Pklr*$^{(246ntdel/246ntdel)}$] do not overexpress the muscle pyruvate kinase isoform (PKM) in RBCs. Immunoblot analysis of RBCs from AS$^{(WT/WT)}$, AS*Pklr*$^{(13ntdel/13ntdel)}$, and AS*Pklr*$^{(246ntdel/246ntdel)}$. While RBCs from mice with *Pklr* null mutations [*Pklr*$^{(13ntdel/13ntdel)}$ or *Pklr*$^{(246ntdel/246ntdel)}$] did not express the RBC pyruvate kinase (PKR) isoform, the expression of PKM was unaltered. (N=3 per genotype).

Supplemental Figure 3

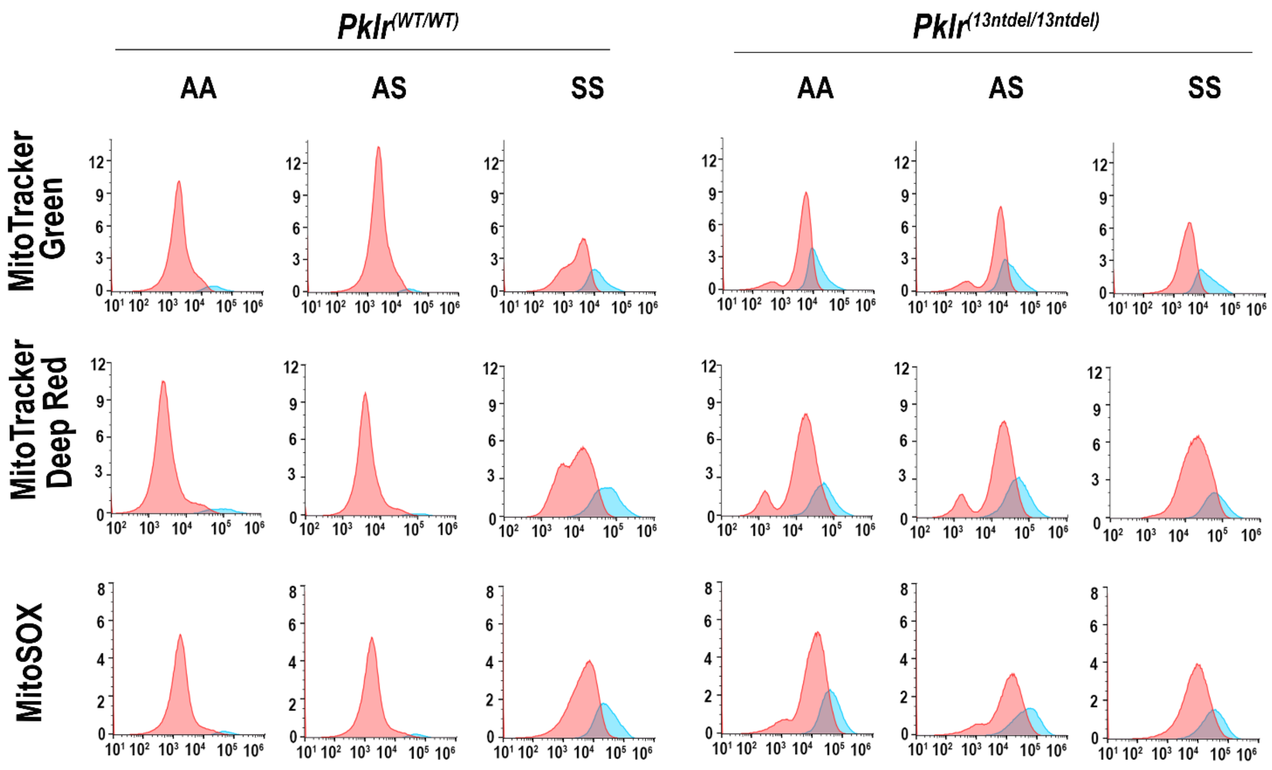

Supplemental Figure 3. Co-inheritance of *Pklr* null mutation [*Pklr*$^{(13ntdel/13ntdel)}$] specific for the RBC pyruvate kinase (PKR) isoform alters mitochondria mass, polarization, and superoxide production in circulating immature (predominantly reticulocytes) and mature RBCs from AA, AS, and SS mice. Representative histograms of RBC mitochondrial staining with MitoTracker Green (mitochondria mass), MitoTracker Deep Red (mitochondria polarization), and MitoSOX (superoxide production) in immature (TER119^{+} and CD71^{high}, which are predominantly reticulocytes, blue curves) and mature (TER119^{+} and CD71^{low}, pink curves) circulating RBCs from AA, AS, and SS mice without [*Pklr* $^{(WT/WT)}$] or with *Pklr* null mutation [*Pklr*$^{(13ntdel/13ntdel)}$]. RBC mitochondrial staining and corresponding histograms were obtained from at least 5 mice for each genotype.

Supplemental Figure 4

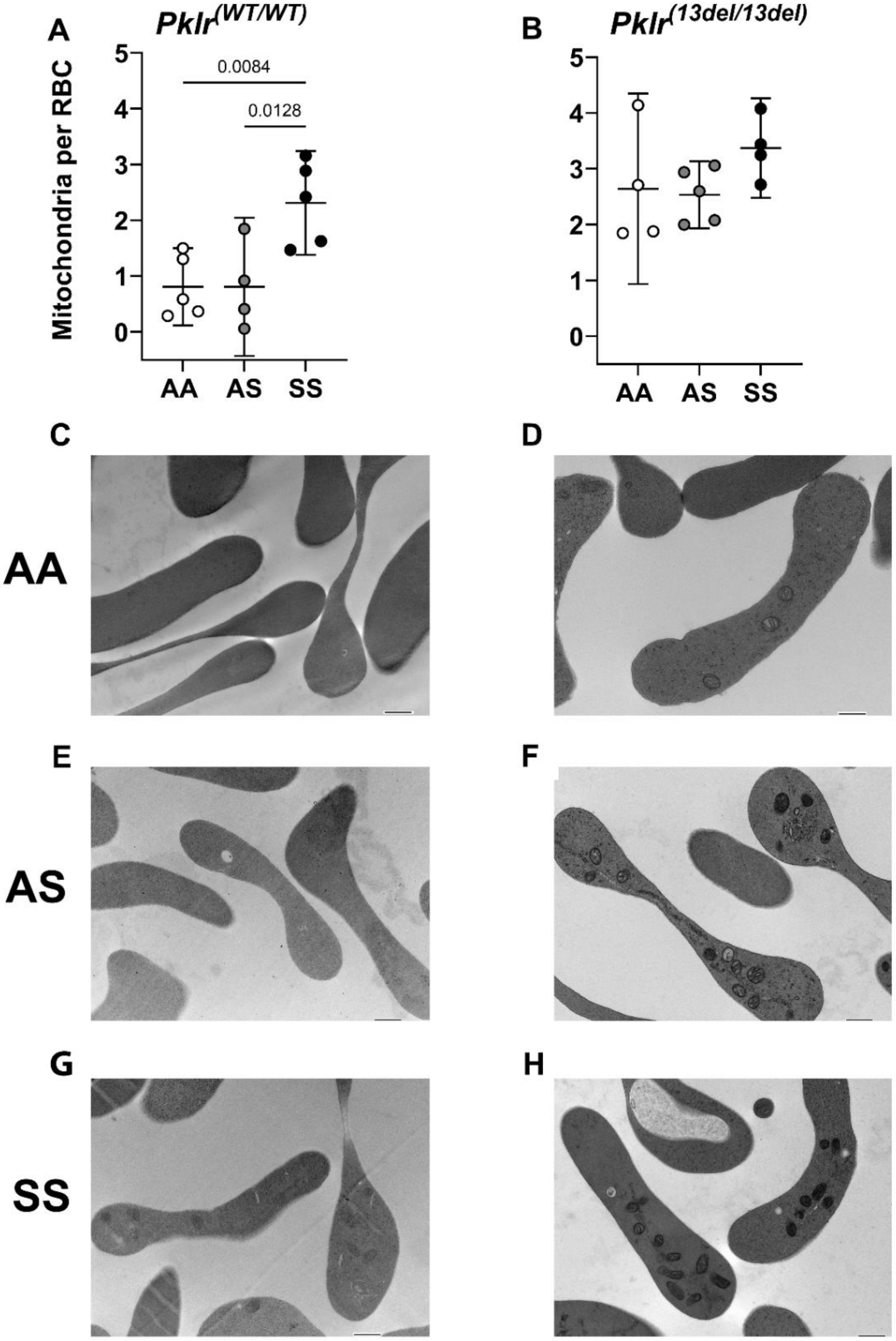

Supplemental Figure 4. **Co-inheritance of *Pklr* null mutation [*Pklr*$^{(13ntdel/13ntdel)}$] specific for**

the RBC pyruvate kinase isoform (PKR) increases mitochondria retention in circulating RBCs from AA, AS, and SS mice. Data are shown as scatter dot plots illustrating individual mouse measurements, with overlaid bars representing the least-squares mean ± 95% confidence intervals. Data were analyzed using a two-way ANOVA, and p values were adjusted for multiple comparisons using the Tukey method (**A** and **B**). Investigators, blinded to animal genotype, analyzed eight whole blood TEM images per mouse. Within these images, individual, anuclear cells with a curved, elliptical shape were classified as mature RBCs. Intracellular organelles with a double-membrane and internal cristae were categorized as mitochondria. For each of the eight images per mouse, the number of mitochondria within all visible mature RBC were quantified and the average number of mitochondria per RBC for each mouse calculated as described (2, 10). In **A** and **B**, each dot represents the average number of mitochondria per RBC per mouse of the indicated genotype. Overall, SS mice had more mitochondria per RBC compared to AA and AS mice ($p<0.0032$, for overall sickle genotype effect, **A** and **B**). Overall, across sickle genotypes, mice with $Pklr^{(13ntdel/13ntdel)}$ mutation had a higher average number of mitochondria per RBC compared to $Pklr^{(WT/WT)}$ mice ($p<0.0001$, for overall *Pklr* mutation effect, **A** and **B**). Post hoc analyses revealed that PKR-deficient AA, AS, and SS mice had a higher average number of mitochondria per RBC compared with their respective $Pklr^{(WT/WT)}$ counterpart ($p=0.0010$, $p=0.0016$, and $p=0.0378$ respectively (**A** and **B**). **C**, **E**, and **G** show representative TEM RBC images from AA$Pklr^{(WT/WT)}$, AS$Pklr^{(WT/WT)}$, and SS$Pklr^{(WT/WT)}$ mice. **D**, **F**, and **H** display representative TEM RBC images from AA$Pklr^{(13ntdel/13ntdel)}$, AS$Pklr^{(13ntdel/13ntdel)}$, and SS$Pklr^{(13ntdel/13ntdel)}$ mice. RBC TEM images were obtained from at least 4 mice per each genotype shown.

Supplemental Figure 5

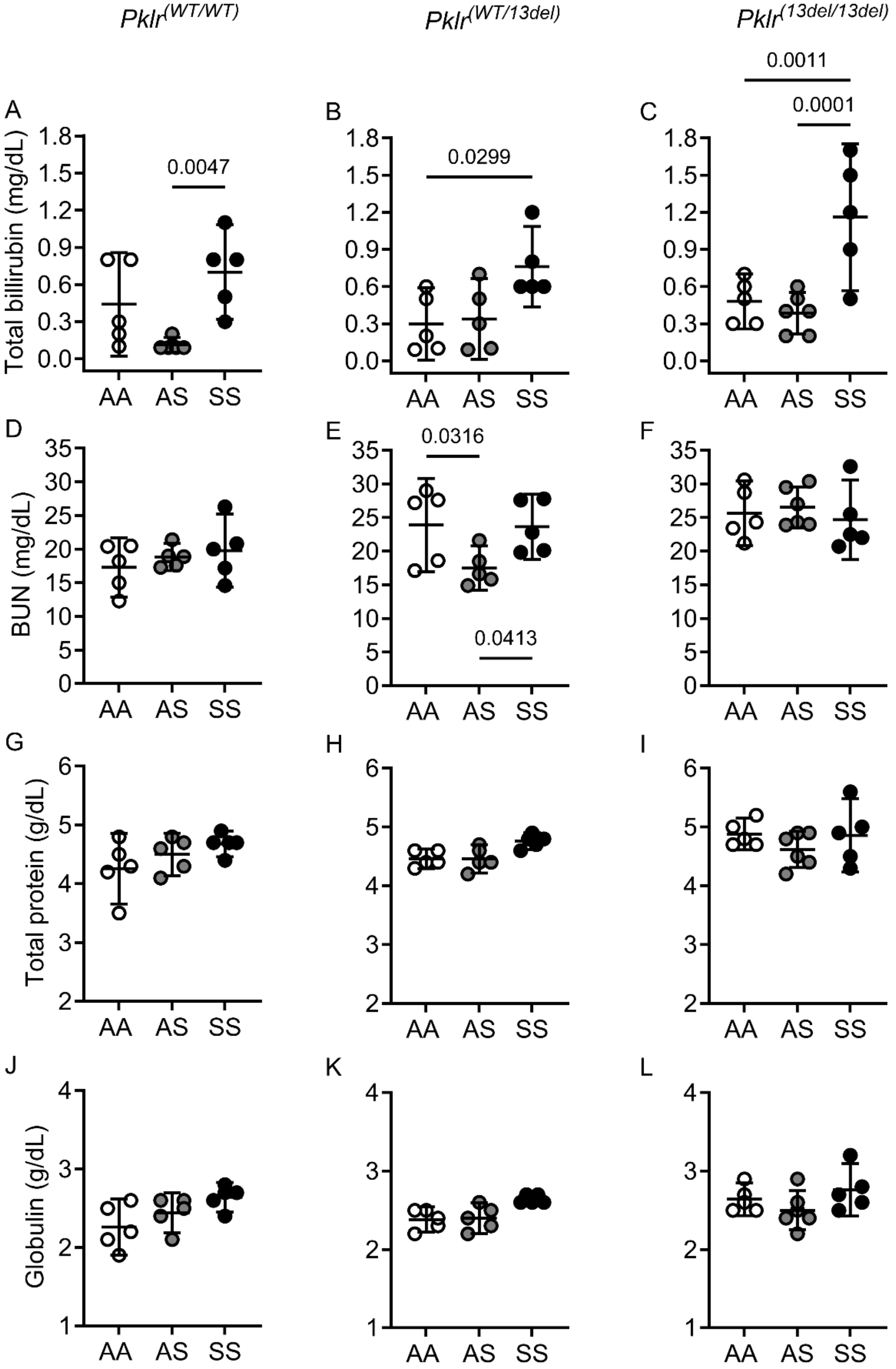

Supplemental Figure 5. Co-inheritance of *Pklr* null mutation [*Pklr*$^{(13ntdel/13ntdel)}$] specific for the RBC pyruvate kinase isoform (PKR) differentially alters biochemical parameters in

AA, AS, and SS Townes mice. Data are shown as scatter dot plots illustrating individual mouse measurements, with overlaid bars representing the least-squares mean ± 95% confidence intervals. Data were analyzed using a two-way ANOVA, and p values were adjusted for multiple comparisons using the Tukey method. All experimental groups included a balanced number of age- and sex-matched mice. Overall, SS mice had higher total bilirubin plasma levels compared to AA and AS mice (p<0.001, **A-C**). Additionally, mice with PKR deficiency [*Pklr* $^{(13ntdel/13ntdel)}$] had higher bilirubin and BUN plasma levels compared with *Pklr*$^{(WT/WT)}$ animals (p=0.0299, p<0.0001, for overall *Pklr* mutation effect respectively, **A-F**). Additionally, AA*Pklr*$^{(13ntdel/13ntdel)}$ had higher protein (**I**) and globulin (**L**) levels compared to AA*Pklr*$^{(WT/WT)}$ (**G** and **J**). Co-inheritance of P*klr*$^{(WT/13ntdel)}$ mutation did not affect blood biochemical parameters in AA, AS, and SS Townes mice (**B**, **E**, **H**, **K**).

Supplemental Figure 6

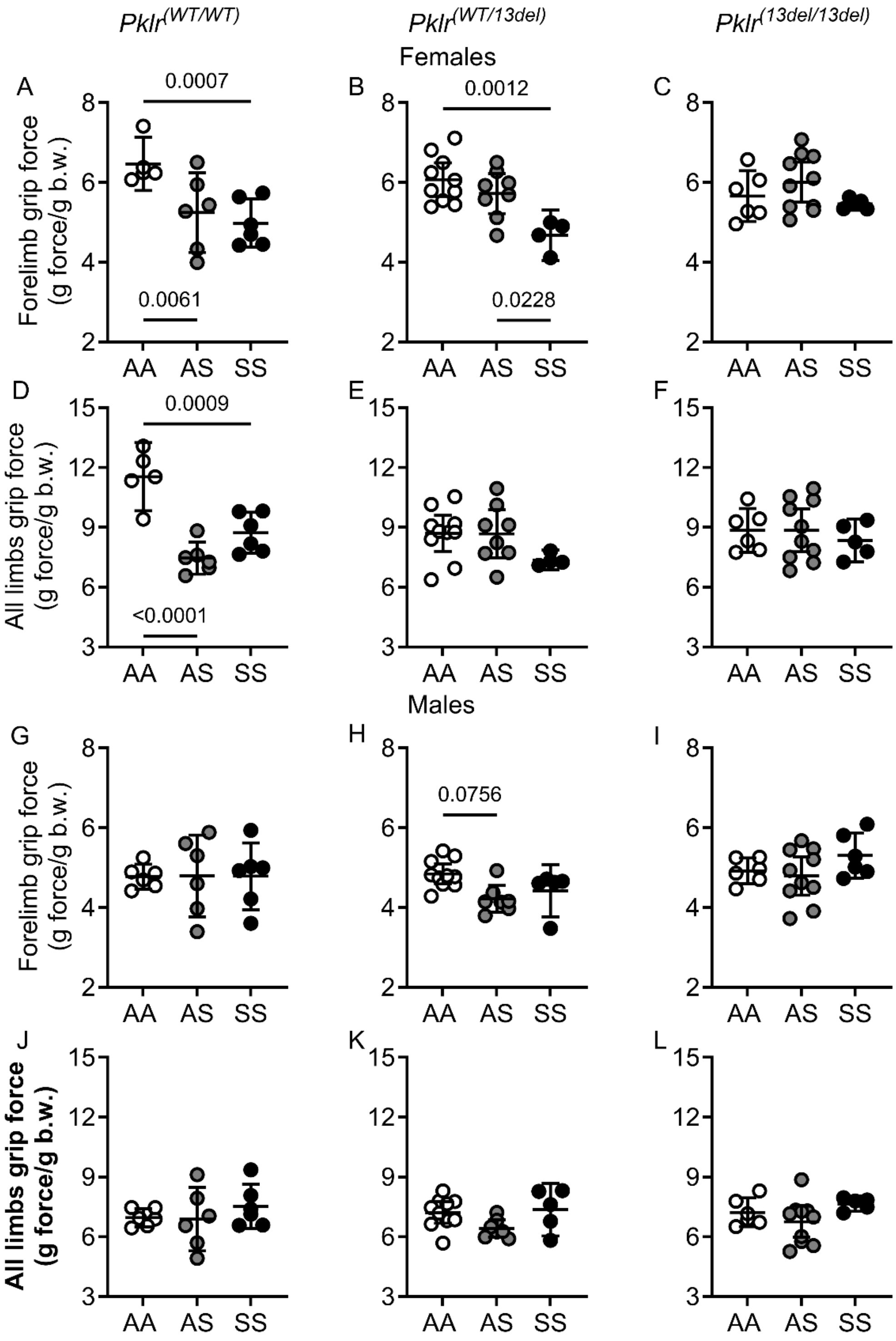

Supplemental Figure 6. Co-inheritance of *Pklr* null mutation [*Pklr*$^{(13ntdel/13ntdel)}$] specific for the RBC pyruvate kinase isoform (PKR) differentially alter grip force in AA, AS, and SS

Townes. Data are shown as scatter dot plots illustrating individual mouse measurements, with overlaid bars representing the least-squares mean ± 95% confidence intervals. Data were analyzed using a two-way ANOVA, and p values were adjusted for multiple comparisons using the Tukey method. All experimental groups included a balanced number of age- and sex-matched mice. Overall, controlling for *Pklr* mutations and sickle genotype, females exhibited greater forelimb and all limbs grip force compared to males (p<0.0001, **A**-**L**). Additionally, among females, SS*Pklr*$^{(WT/WT)}$ and AS*Pklr*$^{(WT/WT)}$ mice had lower forelimb (**A**) and all limbs grip force (**D**) compared with AA *Pklr*$^{(WT/WT)}$. AA*Pklr*$^{(13ntdel/13ntdel)}$ females displayed a trend toward lower forelimb (p=0.0924, **A** and **C**) and lower all-limbs (p=0.0015**, D** and **F**) grip force compared with AA*Pklr*$^{(WT/WT)}$. Conversely, AS*Pklr*$^{(13ntdel/13ntdel)}$ females showed trends towards greater forelimb (p=0.0555, **A** and **C**) and all-limbs grip force (p=0.0720, **D** and **E**) compared with AS*Pklr*$^{(WT/WT)}$. Among SS mice, loss-of- function *Pklr* mutations yielded no significant changes on grip force (**C**, **F**, **I**, **L**). Co-inheritance of P*klr*$^{(WT/13ntdel)}$ mutation did not affect grip force in AA, AS, and SS Townes mice.

Supplemental Figure 7

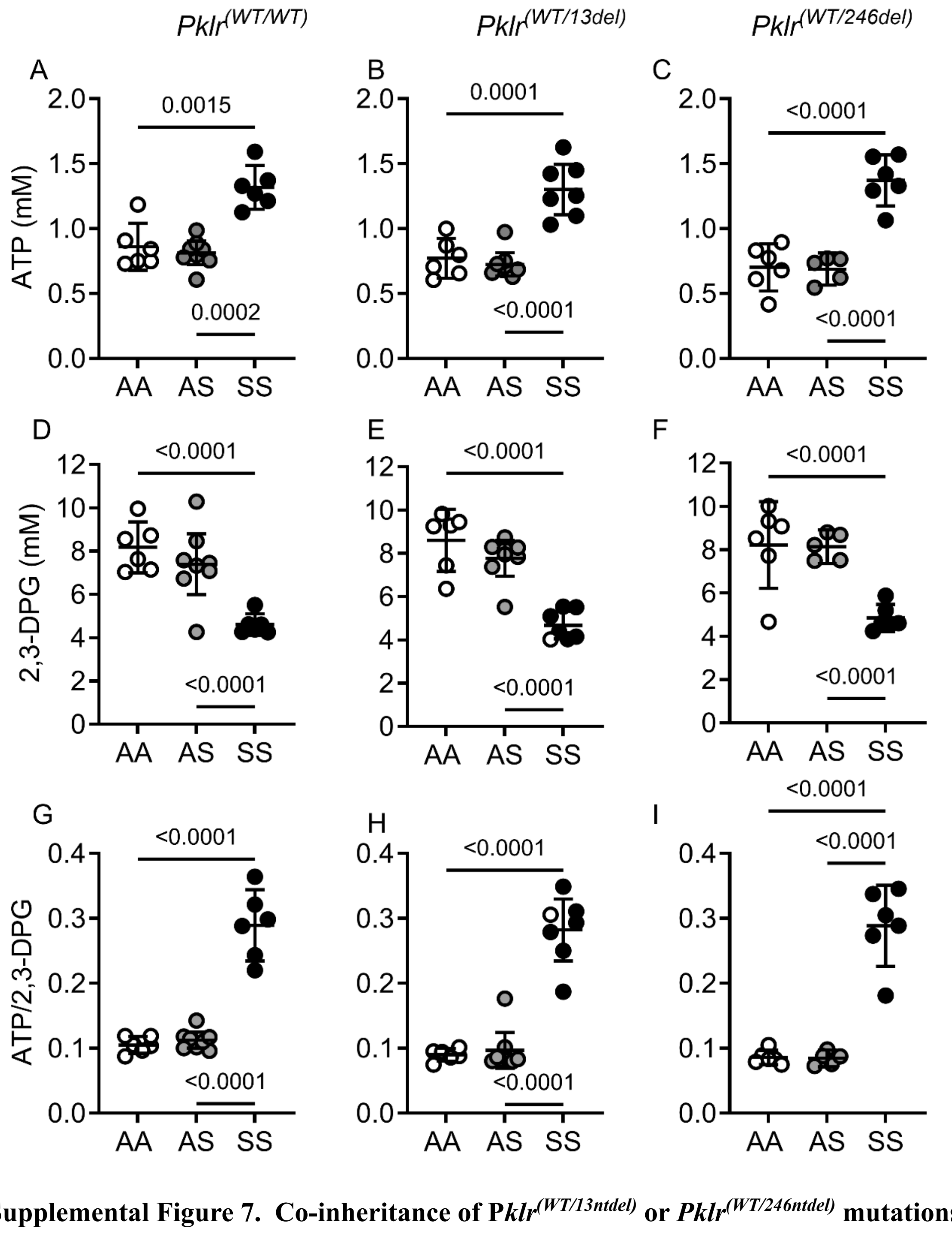

Supplemental Figure 7. Co-inheritance of P*klr*$^{(WT/13ntdel)}$ or *Pklr*$^{(WT/246ntdel)}$ mutations does not affect blood ATP or 2,3-diphosphoglycerate (2,3-DPG) levels in AA, AS, or SS mice. Data are shown as scatter dot plots illustrating individual mouse measurements, with overlaid

bars representing the least-squares mean ± 95% confidence intervals. Data were analyzed using a two-way ANOVA, and p values were adjusted for multiple comparisons using the Tukey method. All experimental groups included a balanced number of age- and sex-matched mice. Mice with *Pklr*$^{(WT/WT)}$ were shown in Figure 1 and are shown here for clarity. AA, AS, and SS with co-inheritance of *Pklr*$^{(WT/13ntdel)}$ or *Pklr*$^{(WT/246ntdel)}$ mutations had similar ATP (**A**-**C**) and 2,3-DPG (**D**-**F**) levels and ATP/2,3-DPG ratios compared to their respective *Pklr*$^{(WT/WT)}$ controls (**G**-**I**).

Supplemental Figure 8

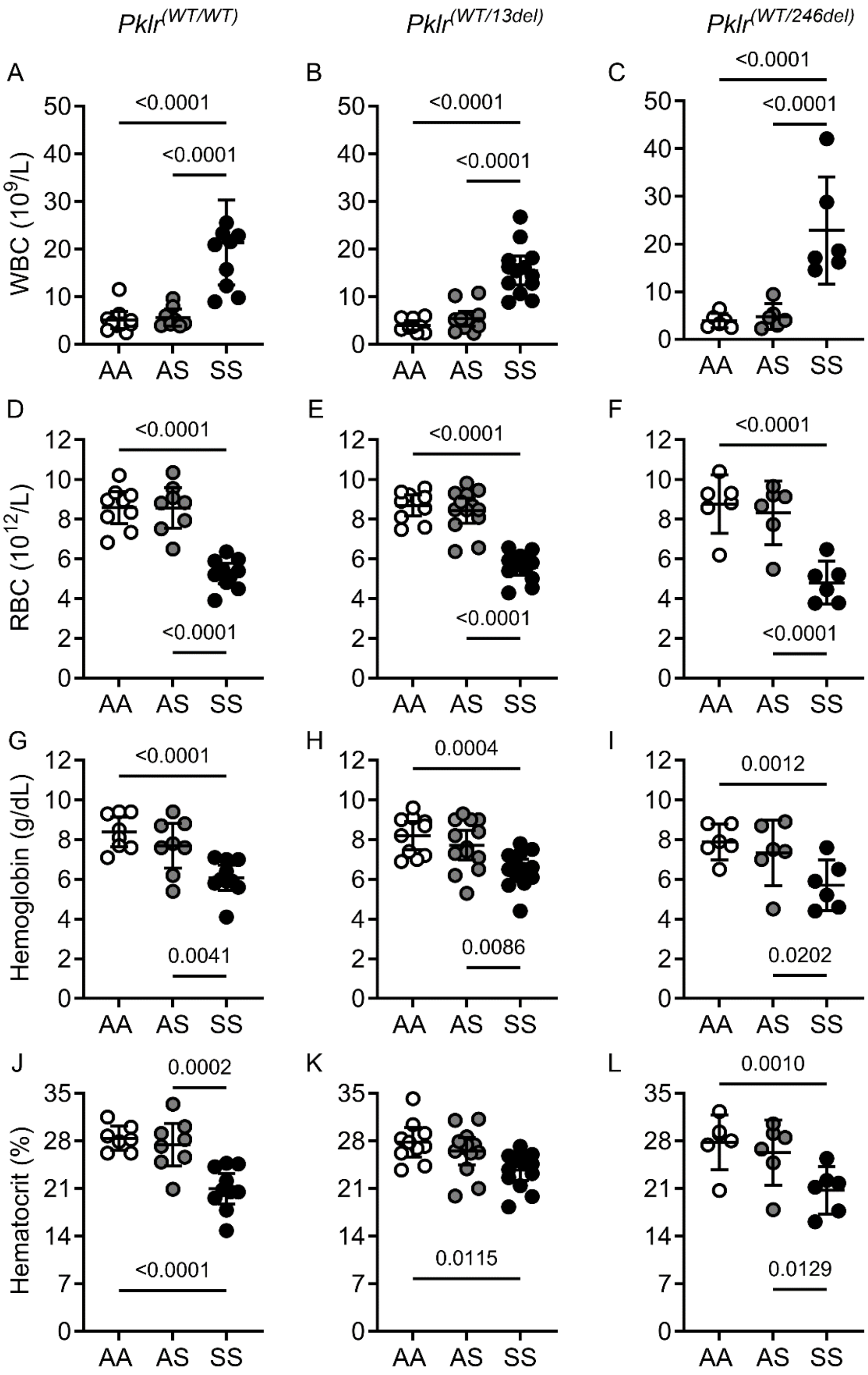

Supplemental Figure 8. Co-inheritance of P*klr*$^{(WT/13ntdel)}$ or *Pklr*$^{(WT/246ntdel)}$ mutations does not affect blood cell counts in AA, AS, or SS mice. Data are shown as scatter dot plots illustrating individual mouse measurements, with overlaid bars representing the least-squares mean ± 95% confidence intervals. Data were analyzed using a two-way ANOVA, and p values were adjusted for multiple comparisons using the Tukey method. All experimental groups included balanced number of age- and sex-matched mice. Mice with *Pklr*$^{(WT/WT)}$ were shown in Figure 2 and are shown here for clarity. AA, AS, and SS with co-inheritance of *Pklr*$^{(WT/13ntdel)}$ or *Pklr*$^{(WT/246ntdel)}$ mutations had similar WBC (**A**-**C**), RBC (**D** - **F**), hemoglobin (**G**-**I**) and hematocrit (**J**-**L**) compared to AA*Pklr*$^{(WT/WT)}$, AS*Pklr*$^{(WT/WT)}$, and SS*Pklr*$^{(WT/WT)}$ respectively.

Supplemental Figure 9

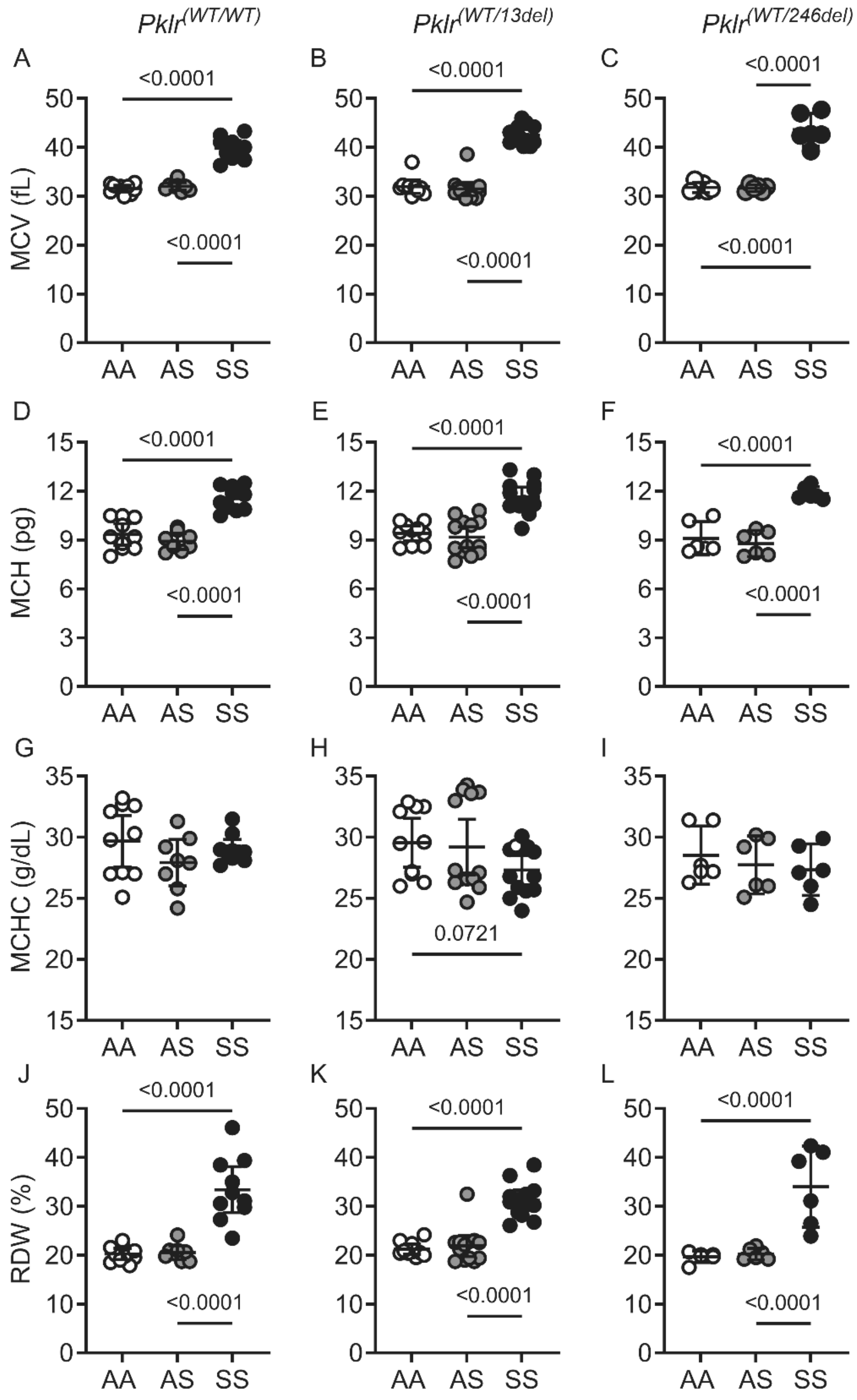

Supplemental Figure 9. Co-inheritance of P*klr*$^{(WT/13ntdel)}$ or *Pklr*$^{(WT/246ntdel)}$ mutations does

not affect hematologic indices in AA, AS, or SS mice. Data are shown as scatter dot plots illustrating individual mouse measurements, with overlaid bars representing the least-squares mean ± 95% confidence intervals. Data were analyzed using a two-way ANOVA, and p values were adjusted for multiple comparisons using the Tukey method. All experimental groups included a balanced number of age- and sex-matched mice. Mice with *Pklr*$^{(WT/WT)}$ were shown in Figure 3 and are shown here for clarity. AA, AS, and SS with co-inheritance of *Pklr*$^{(WT/13ntdel)}$ or *Pklr*$^{(WT/246ntdel)}$ mutations had similar mean corpuscular volume (MCV, **A**-**C**), mean corpuscular hemoglobin (MCH, **D**-**F**), mean corpuscular hemoglobin concentration (MCHC, **G**-**I**), and red cell distribution width (**J**-**L**) compared to AA*Pklr*$^{(WT/WT)}$, AS*Pklr*$^{(WT/WT)}$, and SS*Pklr*$^{(WT/WT)}$ respectively.

Supplemental Figure 10

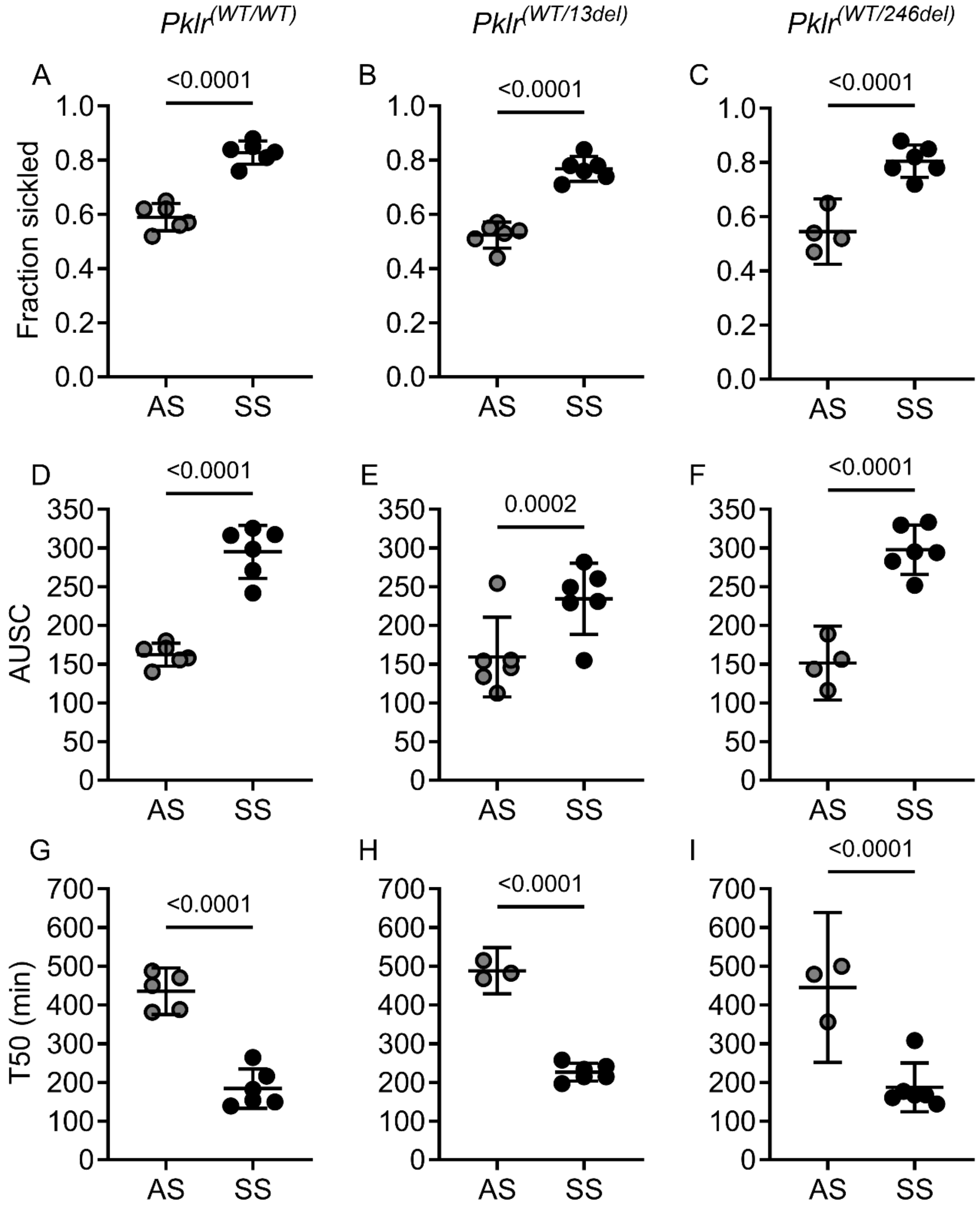

Supplemental Figure 10. Co-inheritance of P*klr*$^{(WT/13ntdel)}$ or *Pklr*$^{(WT/246ntdel)}$ mutations does not alter sickling kinetics in AS or SS mice. Data are shown as scatter dot plots illustrating individual mouse measurements, with overlaid bars representing the least-squares mean ± 95%

confidence intervals. Data were analyzed using a two-way ANOVA, and p values were adjusted for multiple comparisons using the Tukey method. All experimental groups included balanced number of age- and sex-matched mice. Mice with *Pklr*$^{(WT/WT)}$ were shown in Figure 4 and are shown here for clarity. AS and SS mice with co-inheritance of *Pklr*$^{(WT/13ntdel)}$ or *Pklr*$^{(WT/246ntdel)}$ mutations had similar mean fraction of sickled red blood cells (**A**-**C**), area under the sickling curve (AUSC, **D**-**F**) and T50 (time when 50% of the red blood cells are sickled, **G**-**I**) compared to AS*Pklr*$^{(WT/WT)}$and SS*Pklr*$^{(WT/WT)}$ respectively.

Supplemental Figure 11

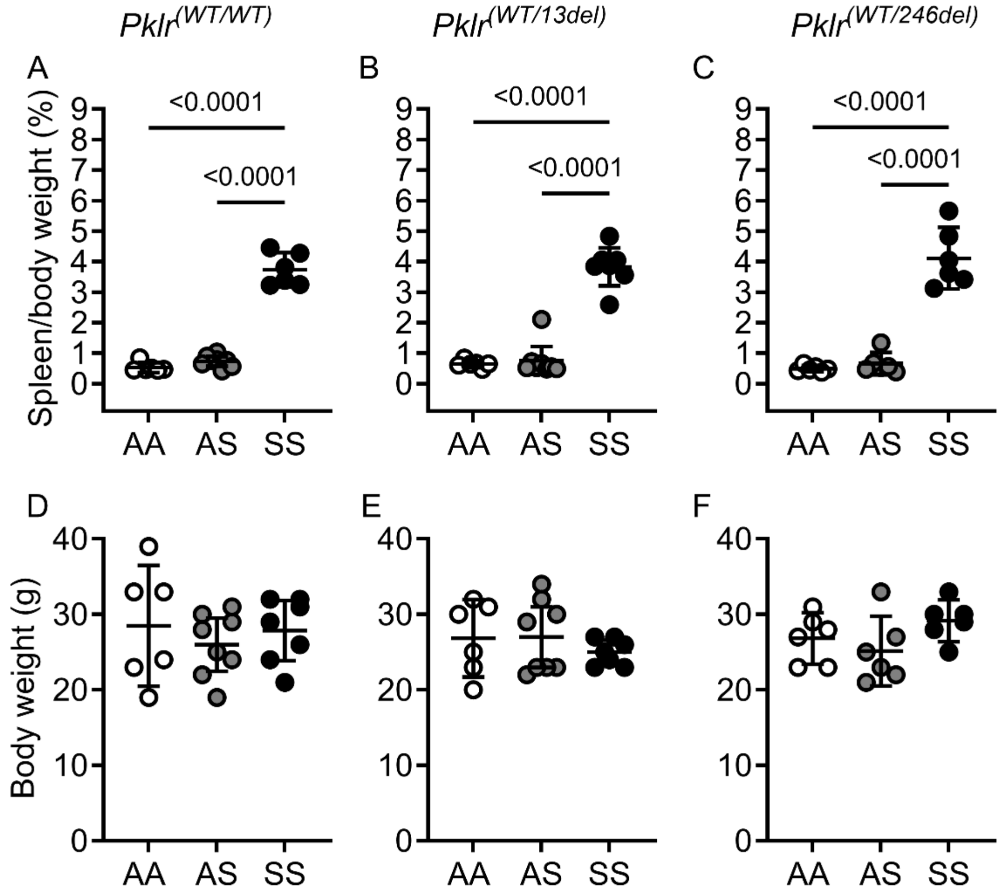

Supplemental Figure 11. Co-inheritance of P*klr*$^{(WT/13ntdel)}$ or *Pklr*$^{(WT/246ntdel)}$ mutations does not affect spleen or body weight in AA, AS, or SS mice. Data are shown as scatter dot plots illustrating individual mouse measurements, with overlaid bars representing the least-squares mean ± 95% confidence intervals. Data were analyzed using a two-way ANOVA, and p values were adjusted for multiple comparisons using the Tukey method. All experimental groups included a balanced number of age- and sex-matched mice. Mice with *Pklr*$^{(WT/WT)}$ were shown in Figure 5 and are shown here for clarity. AA, AS, and SS with co-inheritance of *Pklr*$^{(WT/13ntdel)}$ or *Pklr*$^{(WT/246ntdel)}$ mutations had similar spleen/body weight ratio (**A**-**C**) and body wight (**D**-**F**) compared with AA*Pklr*$^{(WT/WT)}$, AS*Pklr*$^{(WT/WT)}$, and SS*Pklr*$^{(WT/WT)}$ respectively.

Supplemental Figure 12

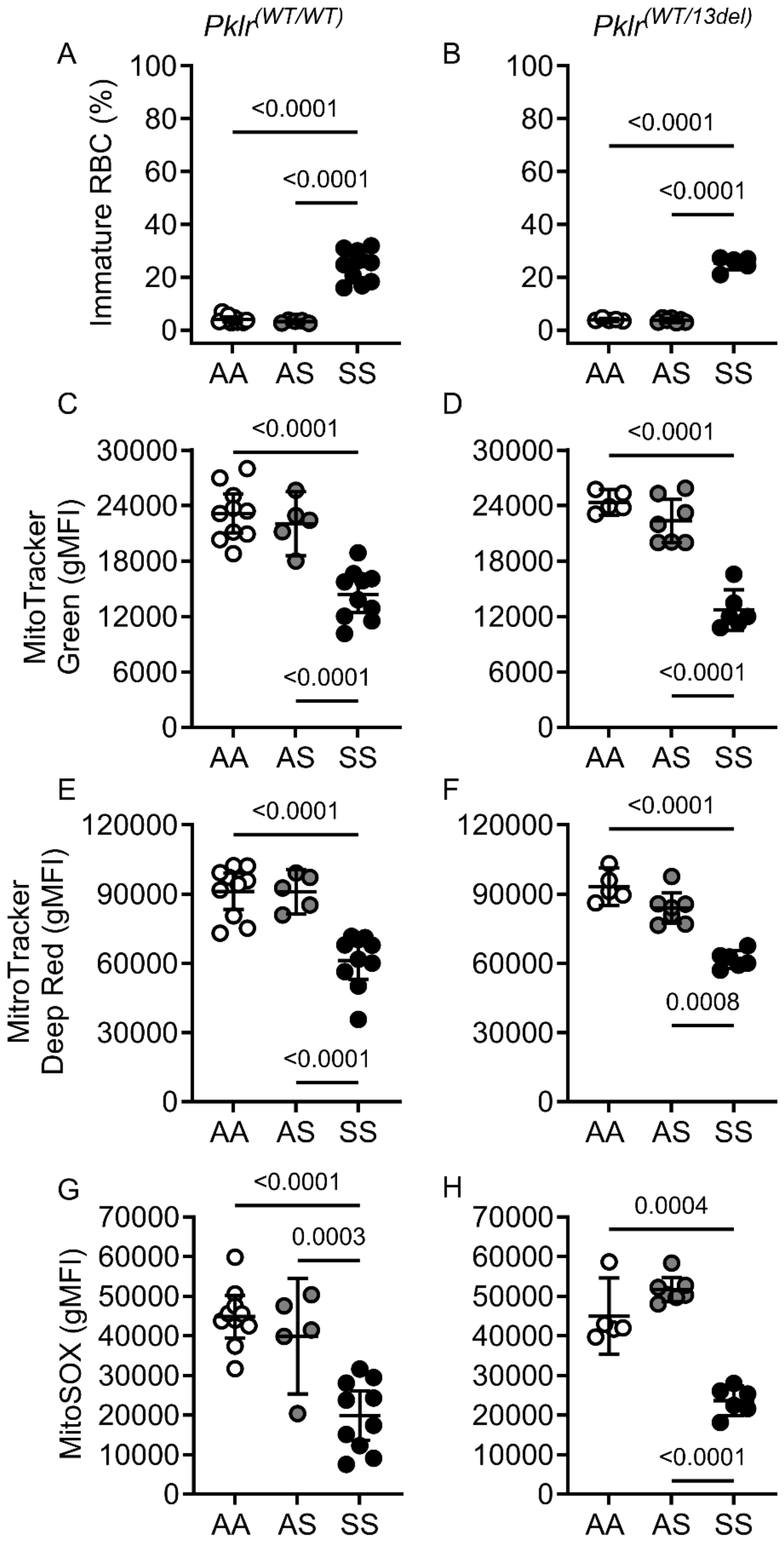

Supplemental Figure 12. Co-inheritance of P*klr*$^{(WT/13ntdel)}$ mutation does not affect

mitochondria content or function in circulating immature red blood cells (RBCs) from AA, AS, or SS mice. Data are shown as scatter dot plots illustrating individual mouse measurements, with overlaid bars representing the least-squares mean ± 95% confidence intervals. Data were analyzed using a two-way ANOVA, and p values were adjusted for multiple comparisons using the Tukey method. All experimental groups included a balanced number of age- and sex-matched mice. Mice with *Pklr*$^{(WT/WT)}$ were shown in Figure 6 and are shown here for clarity. AA, AS, and SS with co-inheritance of *Pklr*$^{(WT/13ntdel)}$ had no significant changes in the percentage of immature RBCs (predominantly reticulocytes) compared with their respective *Pklr*$^{(WT/WT)}$ counterparts (**A** and **B**). Additionally immature RBCS from AA, AS, and SS with co-inheritance of *Pklr*$^{(WT/13ntdel)}$ showed no significant changes in mitochondrial mass (MitoTracker Green, **C** and **D**), mitochondrial membrane polarization (MitoTracker Deep Red, **E** and **F**) or superoxide content (MitoSOX, **G** and **H**) compared to their respective *Pklr*$^{(WT/WT)}$ counterparts.

Supplemental Figure 13

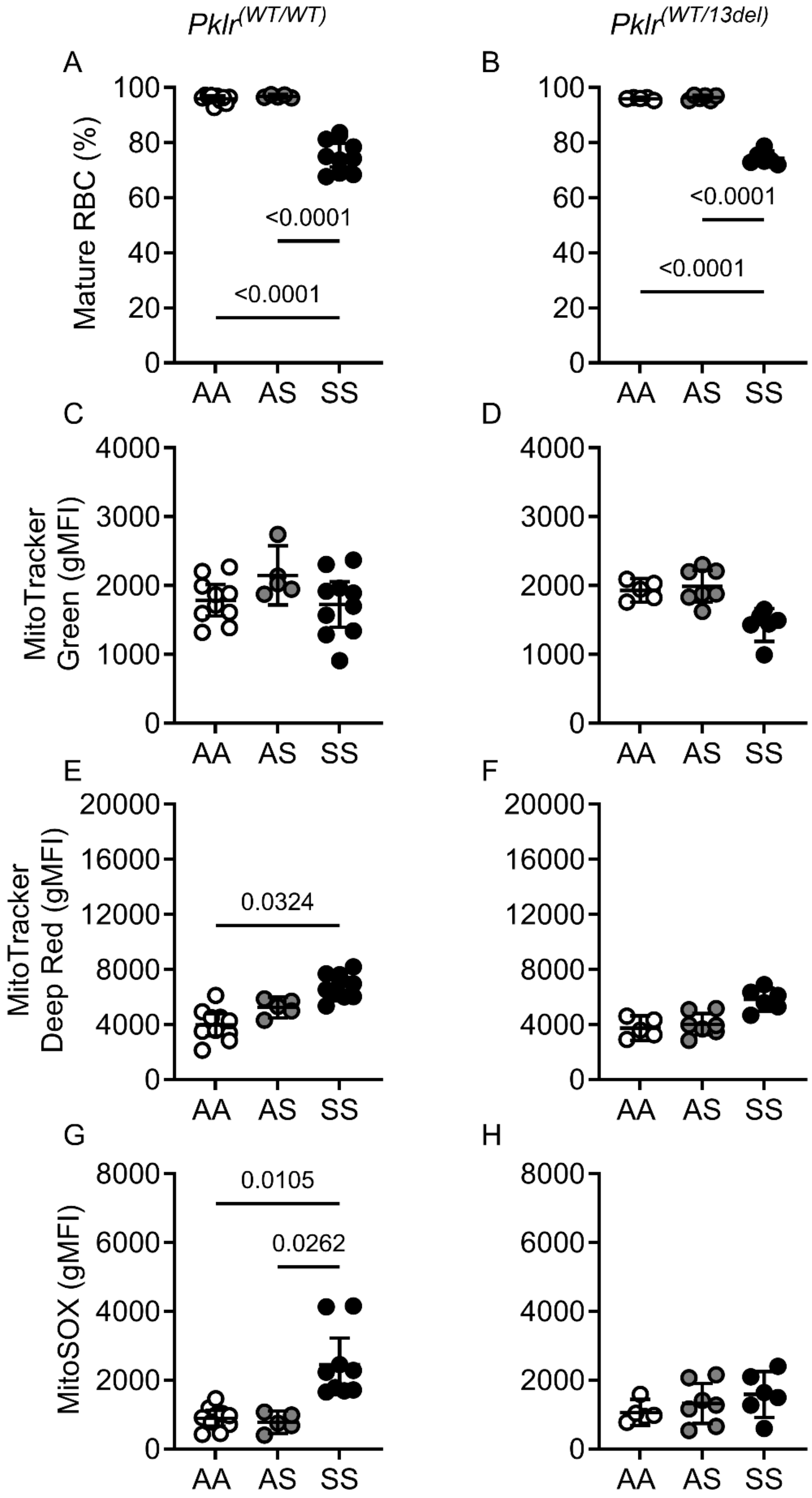

Supplemental Figure 13. Co-inheritance of P*klr*$^{(WT/13ntdel)}$ mutation does not affect

mitochondria content or function in mature red blood cells in AA, AS, or SS mice. Data are shown as scatter dot plots illustrating individual mouse measurements, with overlaid bars representing the least-squares mean ± 95% confidence intervals. Data were analyzed using a two-way ANOVA, and p values were adjusted for multiple comparisons using the Tukey method. All experimental groups included a balanced number of age- and sex-matched mice. Mice with *Pklr*$^{(WT/WT)}$ were shown in Figure 7 and are shown here clarity. AA, AS, and SS with co-inheritance of *Pklr*$^{(WT/13ntdel)}$ had no significant changes in the percentage of mature RBCs (**A** and **B**) compared to their respective *Pklr*$^{(WT/WT)}$ counterparts. Additionally mature RBCS from AA, AS, and SS with co-inheritance of *Pklr*$^{(WT/13ntdel)}$ showed no significant changes in mitochondrial mass (MitoTracker Green, **C** and **D**), mitochondrial membrane polarization (MitoTracker Deep Red, **E** and **F**) or superoxide content (MitoSOX, **G** and **H**) compared to their respective *Pklr*$^{(WT/WT)}$ counterparts.

References

1. Wang H, Yang H, Shivalila CS, Dawlaty MM, Cheng AW, Zhang F, et al. One-step generation of mice carrying mutations in multiple genes by CRISPR/Cas-mediated genome engineering. *Cell.* 2013;153(4):910-8.
2. Quezado ZMN, Kamimura S, Smith M, Wang X, Heaven MR, Jana S, et al. Mitapivat increases ATP and decreases oxidative stress and erythrocyte mitochondria retention in a SCD mouse model. *Blood Cells Mol Dis.* 2022;95:102660.
3. Kim H, Kosinski P, Kung C, Dang L, Chen Y, Yang H, et al. A fit-for-purpose LC-MS/MS method for the simultaneous quantitation of ATP and 2,3-DPG in human K(2)EDTA whole blood. *Journal of chromatography B, Analytical technologies in the biomedical and life sciences.* 2017;1061-1062:89-96.
4. Dunkelberger EB, Metaferia B, Cellmer T, and Henry ER. Theoretical Simulation of Red Cell Sickling Upon Deoxygenation Based on the Physical Chemistry of Sickle Hemoglobin Fiber Formation. *J Phys Chem B.* 2018;122(49):11579-90.
5. Metaferia B, Cellmer T, Dunkelberger EB, Li Q, Henry ER, Hofrichter J, et al. Phenotypic screening of the ReFRAME drug repurposing library to discover new drugs for treating sickle cell disease. *Proc Natl Acad Sci U S A.* 2022;119(40):e2210779119.
6. Mozzarelli A, Hofrichter J, and Eaton WA. Delay time of hemoglobin S polymerization prevents most cells from sickling in vivo. *Science.* 1987;237(4814):500-6.
7. Monaco G, Chen H, Poidinger M, Chen J, de Magalhães JP, and Larbi A. flowAI: automatic and interactive anomaly discerning tools for flow cytometry data. *Bioinformatics.* 2016;32(16):2473-80.

8. Khaibullina A, Almeida LEF, Kamimura S, Zerfas PM, Smith ML, Vogel S, et al. Sickle cell disease mice have cerebral oxidative stress and vascular and white matter abnormalities. *Blood Cells Mol Dis.* 2021;86:102493.

9. Almeida LEF, Wang L, Kamimura S, Zerfas PM, Smith ML, Neto OLA, et al. Locomotor mal-performance and gait adaptability deficits in sickle cell mice are associated with vascular and white matter abnormalities and oxidative stress in cerebellum. *Brain Res.* 2020;1746:146968.

10. Sbardella D, Tundo GR, Campagnolo L, Valacchi G, Orlandi A, Curatolo P, et al. Retention of Mitochondria in Mature Human Red Blood Cells as the Result of Autophagy Impairment in Rett Syndrome. *Scientific reports.* 2017;7(1):12297.

11. Calhoun G, Wang L, Almeida LE, Kenyon N, Afsar N, Nouraie M, et al. Dexmedetomidine ameliorates nocifensive behavior in humanized sickle cell mice. *Eur J Pharmacol.* 2015;754:125-33.

12. Wang L, Almeida LEF, de Souza Batista CM, Khaibullina A, Xu N, Albani S, et al. Cognitive and behavior deficits in sickle cell mice are associated with profound neuropathologic changes in hippocampus and cerebellum. *Neurobiol Dis.* 2016;85:60-72.
