# Supplementary material for: Pyruvate kinase deficiency modifies sickle hemoglobin carrier and sickle cell disease phenotypes in mice
Source: JCI Insight. 2026 Jan 8;11(4):e195682. doi: 10.1172/jci.insight.195682 (PMC12956008; doi:10.1172/jci.insight.195682)

Supplemental Figure 1B, AA

RBC PKR immunoblot

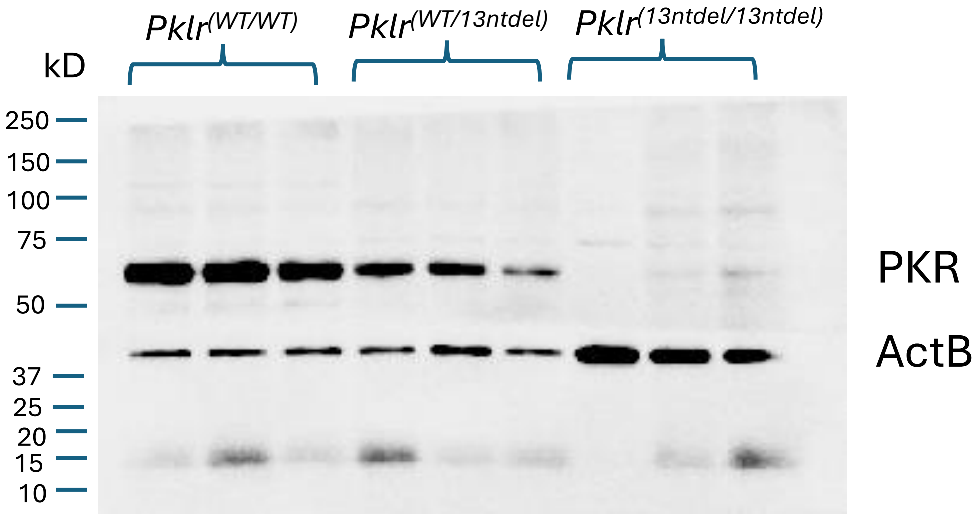

Supplemental Figure 1B, AA

Liver PKL immunoblot

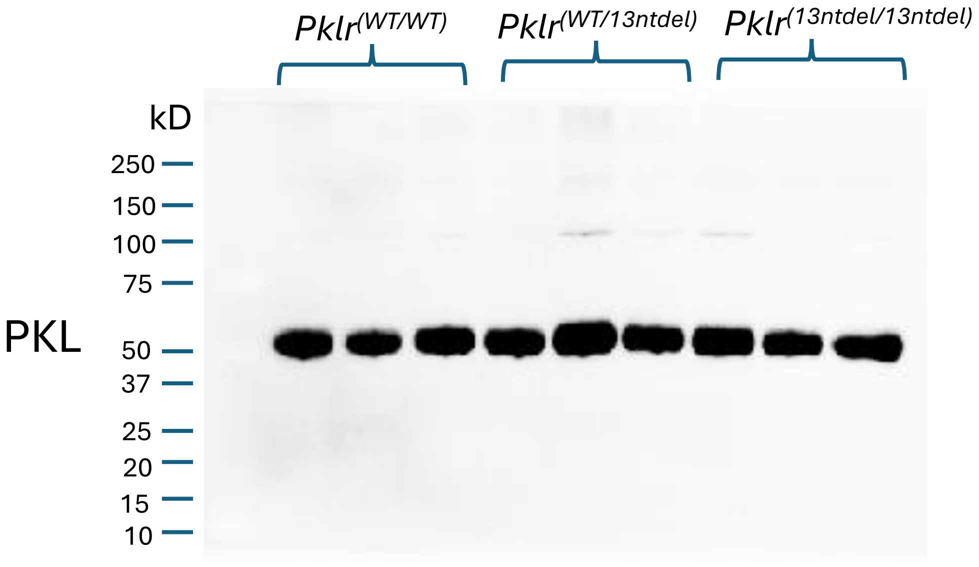

Liver ActB immunoblot

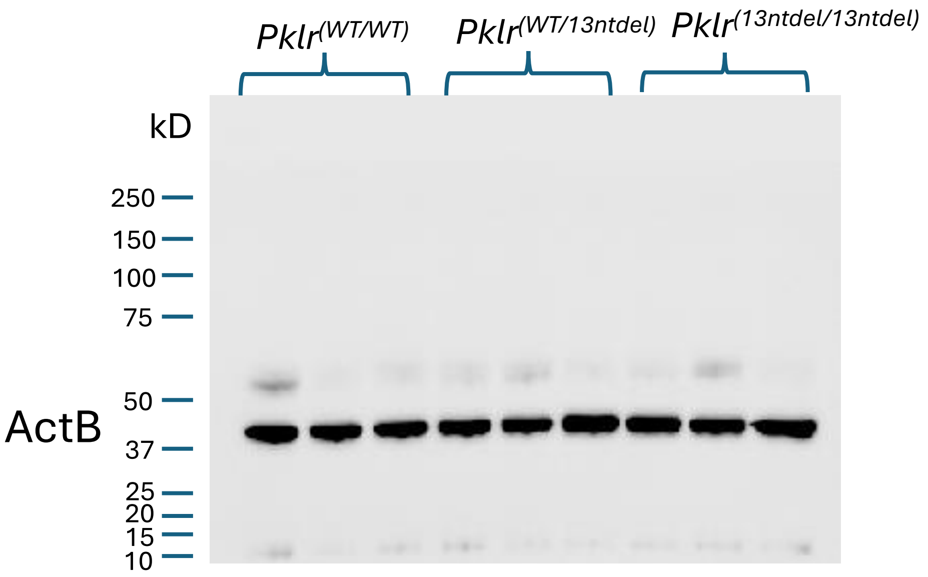

Probed with anti-PKL first, then deprived the filter, probed with anti-ActB. PKL still showed weak signal

Supplemental Figure 1C, AS

RBC PKR immunoblot

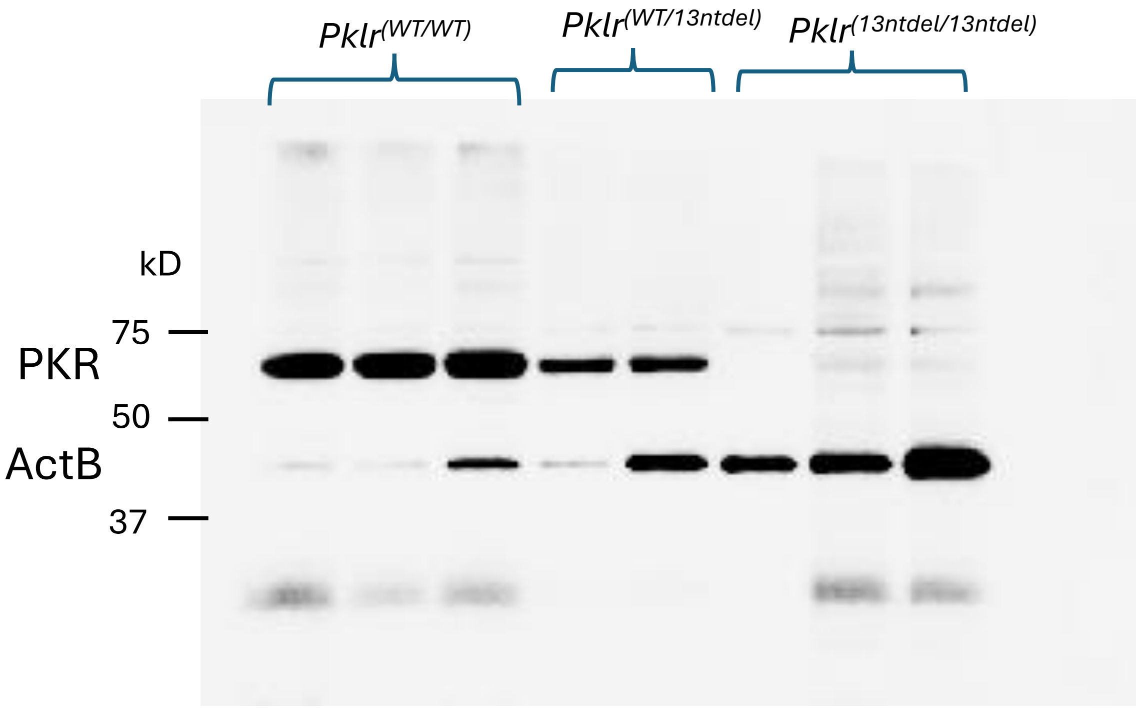

Probed with anti-PKR and anti-ActB

original

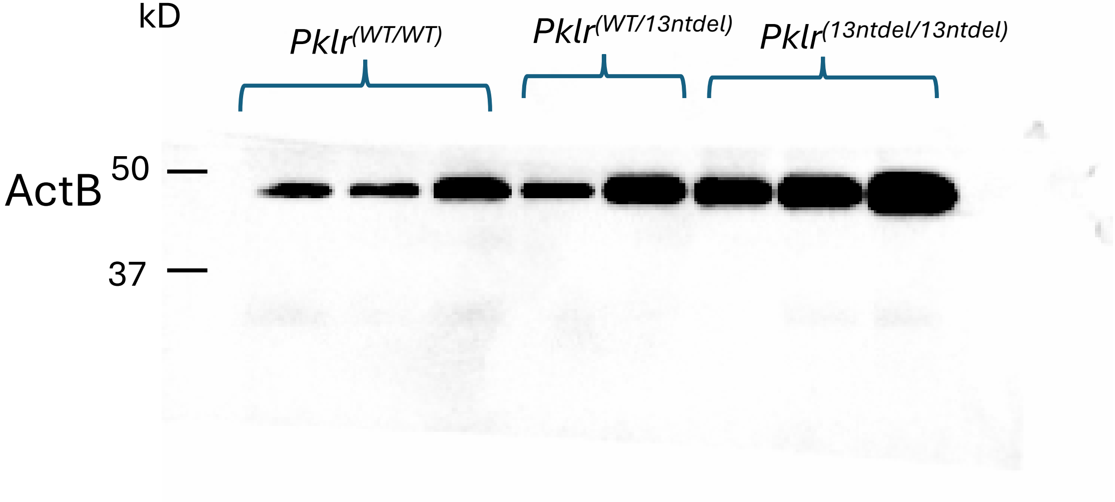

Cut the filter, probe with anti-ActB

Supplemental Figure 1C, AS

Liver PKL immunoblot

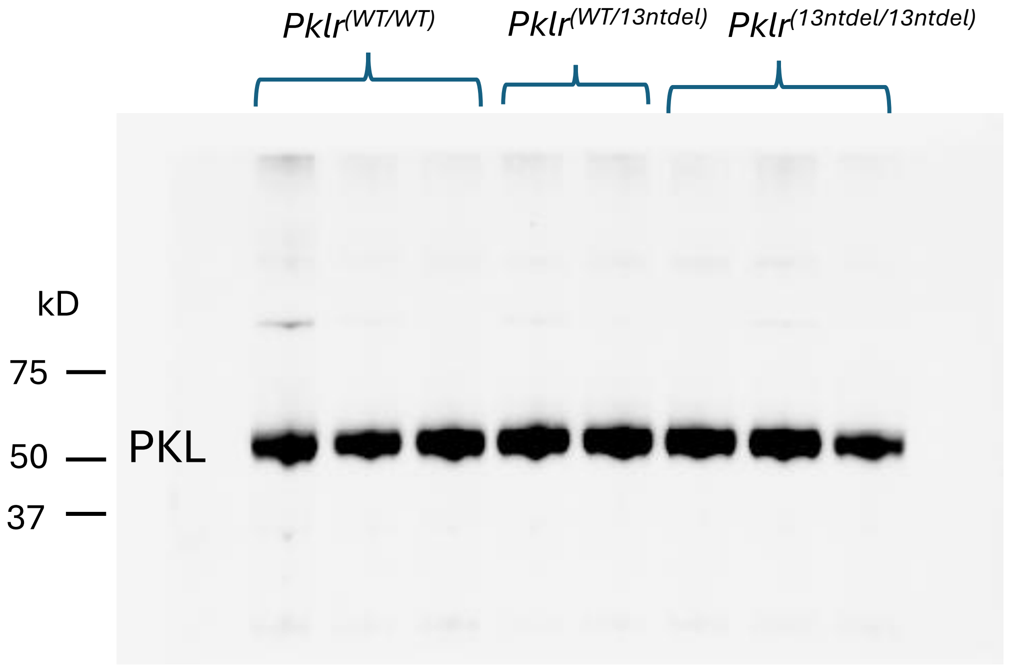

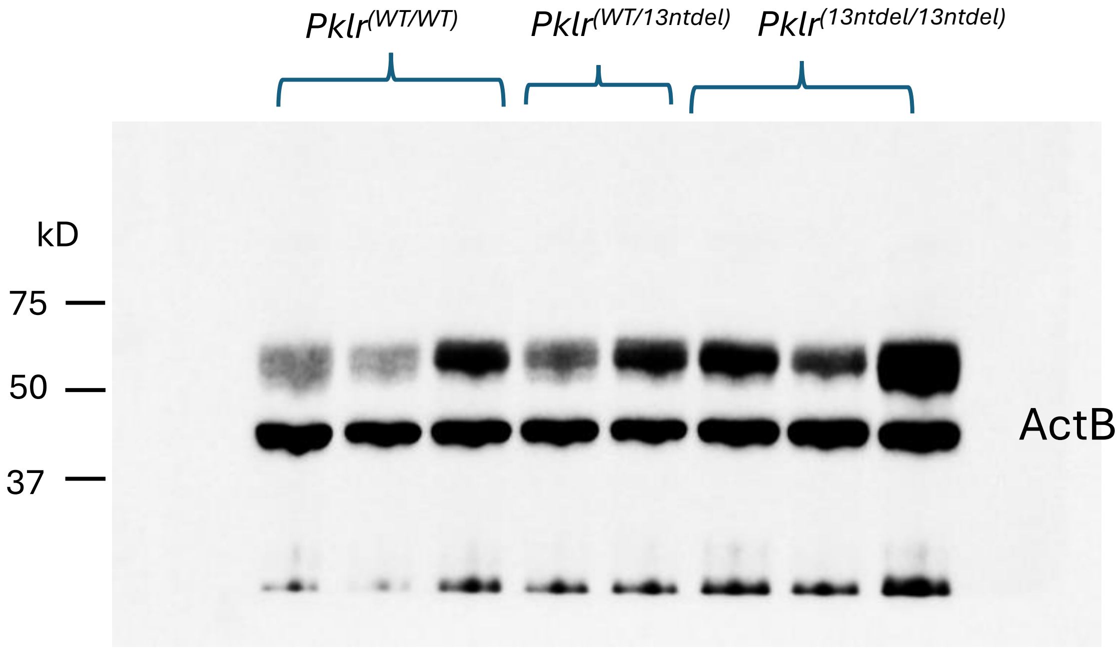

Probed with anti-PKL first, then deprived the filter, probed with anti-ActB. PKL still showed weak signal

Supplemental Figure 1D, SS

RBC PKR immunoblot

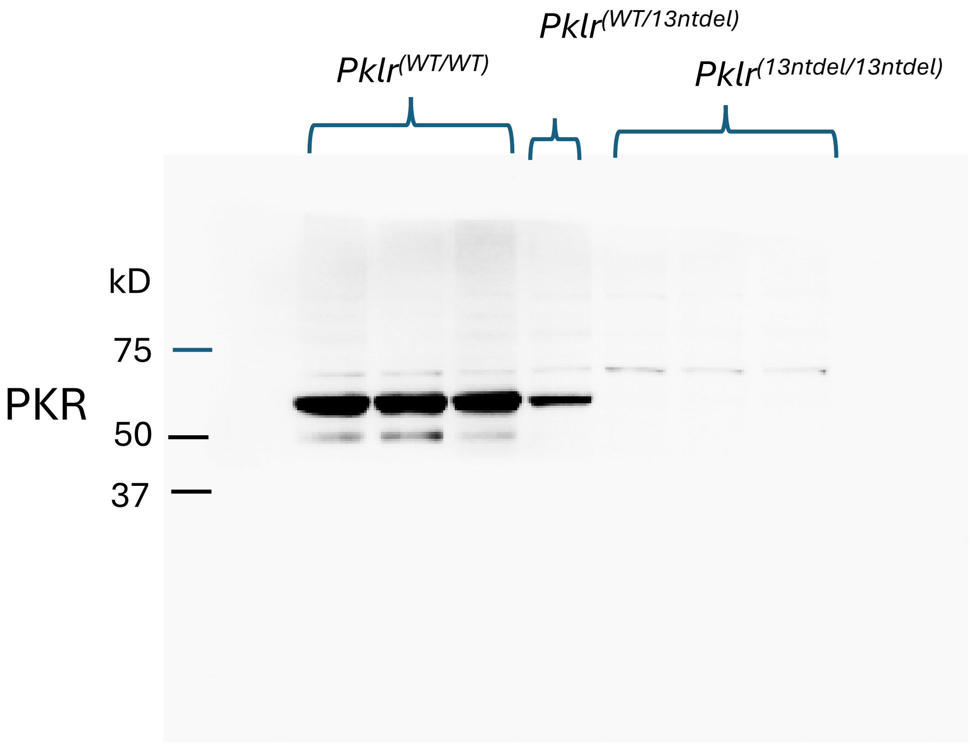

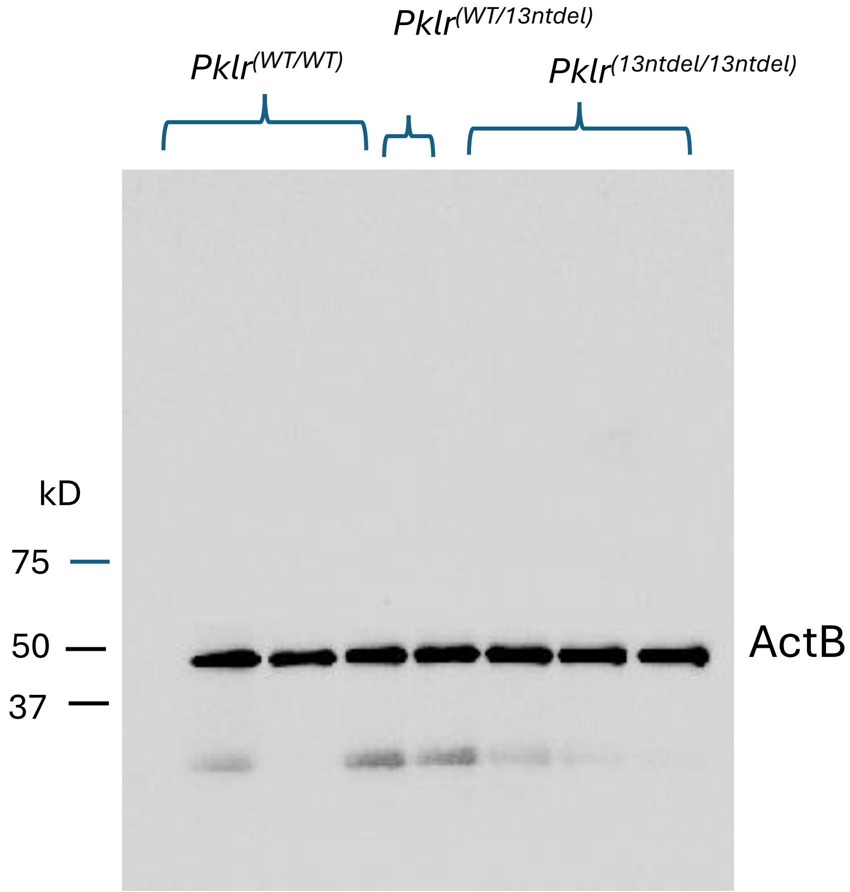

Supplemental Figure 1D, SS

Liver PK immunoblot

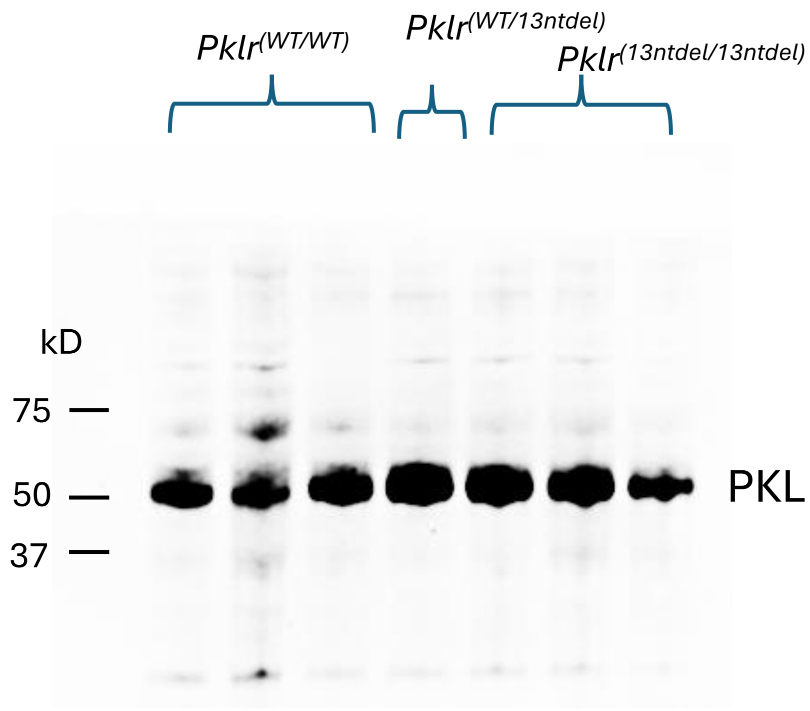

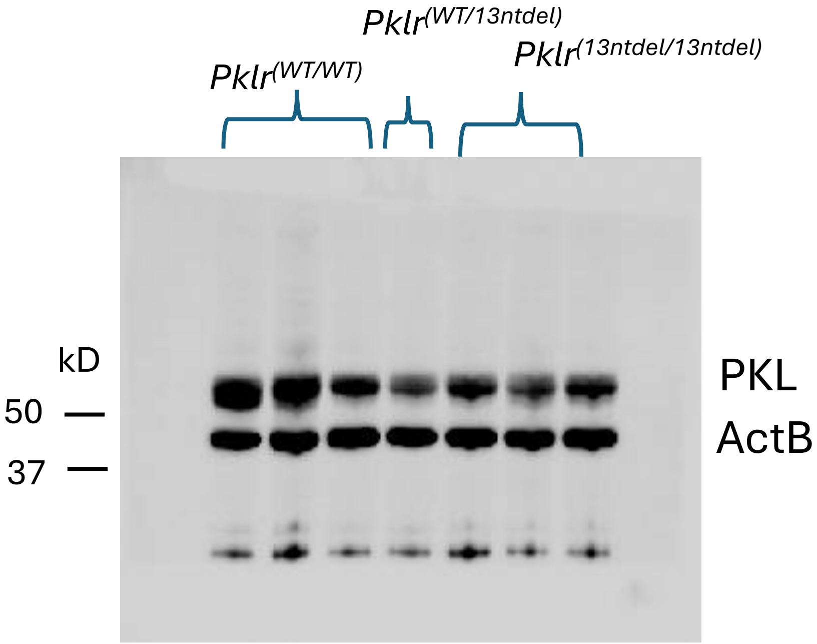

Probed with anti-PKL first, then deprived the filter, probed with anti-ActB. PKL still showed weak signal

Supplemental Figure 2A

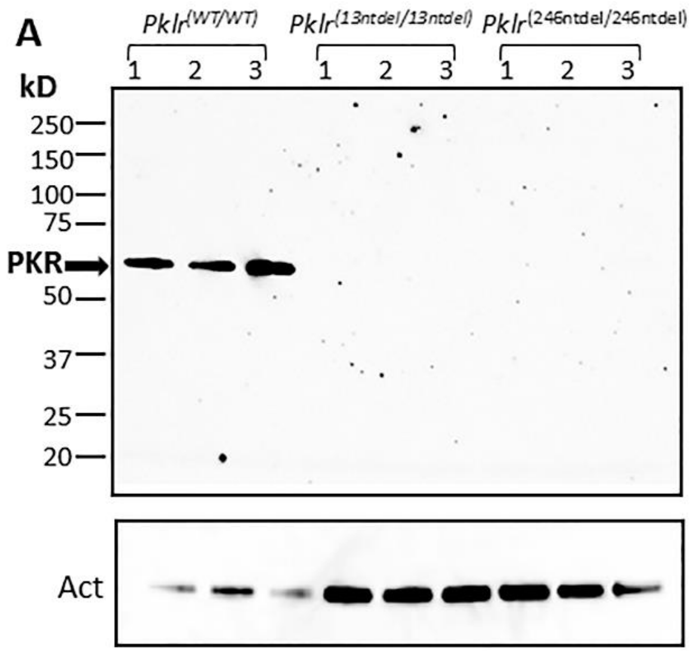

Supplemental Figure 2B

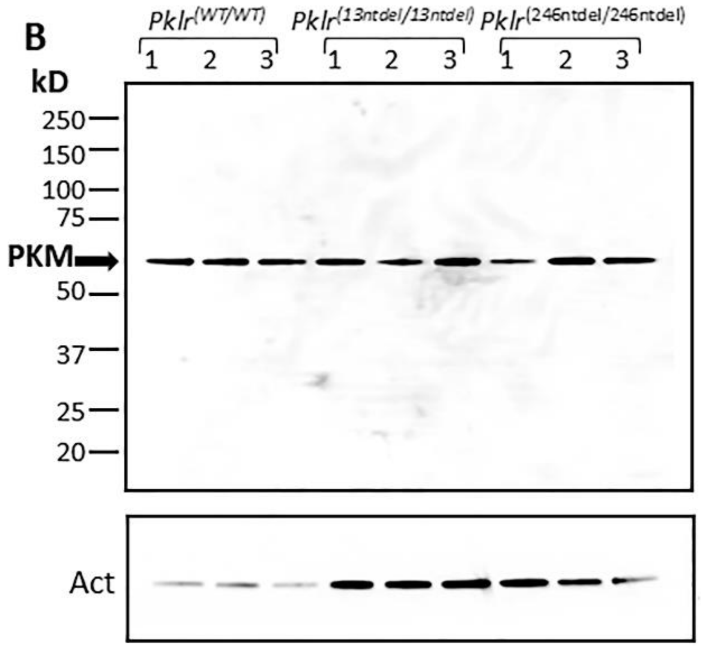

Supplement: Unedited blot and gel images [file jciinsight-11-195682-s058.pdf]
